# Bidirectionally Regulating Viral and Cellular Ferroptosis with Metastable Iron Sulfide Against Influenza Virus

**DOI:** 10.1002/advs.202206869

**Published:** 2023-04-24

**Authors:** Xinyu Miao, Yinyan Yin, Yulian Chen, Wenhui Bi, Yuncong Yin, Sujuan Chen, Daxin Peng, Lizeng Gao, Tao Qin, Xiufan Liu

**Affiliations:** ^1^ College of Veterinary Medicine Yangzhou University Yangzhou Jiangsu 225009 P. R. China; ^2^ Joint International Research Laboratory of Agriculture and Agri‐Product Safety the Ministry of Education of China Yangzhou University Yangzhou Jiangsu 225009 P. R. China; ^3^ College of Medicine Yangzhou University Yangzhou Jiangsu 225009 P. R. China; ^4^ International Research Laboratory of Prevention and Control of Important Animal Infectious Diseases and Zoonotic Diseases of Jiangsu Higher Education Institutions Yangzhou University Yangzhou Jiangsu 225009 P. R. China; ^5^ Guangling College Yangzhou University Yangzhou Jiangsu 225000 P. R. China; ^6^ CAS Engineering Laboratory for Nanozyme Institute of Biophysics Chinese Academy of Sciences Beijing 100101 P. R. China; ^7^ Jiangsu Co‐innovation Center for Prevention and Control of Important Animal Infectious Diseases and Zoonoses Yangzhou University Yangzhou Jiangsu 225009 P. R. China; ^8^ Jiangsu Research Centre of Engineering and Technology for Prevention and Control of Poultry Disease Yangzhou Jiangsu 225009 P. R. China

**Keywords:** ferroptosis, influenza virus, metastable iron sulfides, therapy

## Abstract

Influenza virus with numerous subtypes and frequent variation limits the development of high‐efficacy and broad‐spectrum antiviral strategy. Here, a novel multi‐antiviral metastable iron sulfides (mFeS) against various influenza *A/B* subtype viruses is developed. This work finds that mFeS induces high levels of lipid peroxidation and •OH free radicals in the conservative viral envelope, which depends on Fe^2+^. This phenomenon, termed as a viral ferroptosis, results in the loss of viral infectibility and pathogenicity in vitro and in vivo, respectively. Furthermore, the decoction of mFeS (Dc(mFeS)) inhibits cellular ferroptosis‐dependent intracellular viral replication by correcting the virus‐induced reprogrammed sulfur metabolism, a conserved cellular metabolism. Notably, personal protective equipment (PPE) that is loaded with mFeS provides good antiviral protection. Aerosol administration of mFeS combined with the decoction (mFeS&Dc) has a potential therapeutic effect against H1N1 lethal infection in mice. Collectively, mFeS represents an antiviral alternative with broad‐spectrum activity against intracellular and extracellular influenza virus.

## Graphical abstract

mFeS induces the viral ferroptosis by enhancing the lipid peroxidation level, which results in a failed viral infection. In addition, Dc(mFeS) suppresses the cellular ferroptosis‐dependent intracellular replication of influenza virus via restoring influenza virus‐induced imbalance of sulfur metabolism, and the supply of intracellular S^0^. Comprehensively, mFeS&Dc through aerosol administration shows a potential preventive therapy against H1N1 virus infection in mice.

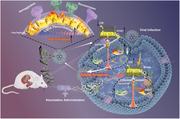

## 1 Introduction

Influenza viruses cause seasonal influenza and annually threaten human health worldwide,^[^ ^1^ ^]^ especially the influenza A virus (IAV) and influenza B virus (IBV). The strain H1N1 of IAV resulted in a pandemic in 2009 that affected more than 214 countries in one and a half years, and caused ≈20 000 deaths.^[^ ^2^ ^]^ Since 2013, an emerging strain of the avian influenza virus (AIV) (H7N9)^[^ ^3^ ^]^ has affected more than 1500 individuals, and caused 600 deaths in China. H5 subtype AIV, especially H5N1, affects more than 800 individuals and results in 400 deaths worldwide,^[^ ^4^ ^]^ and H5N8 subtype AIV has also been demonstrated to be highly pathogenic to mammals in our recent works.^[^ ^5^ ^]^ Although IBV does not cause pandemics, it accounts for a notable proportion of seasonal influenza cases, representing 22%–44% of influenza deaths in children.^[^ ^6^ ^]^ Vaccination is used to limit the spread of influenza.^[^ ^7^ ^]^ Current influenza vaccines offer limited protection due to the high mutability of the virus. Annual updates to the vaccine are required to target the main circulating strains. A high number of mutations occur owing to high antigenic drift on two surface glycoproteins of the influenza virus: hemagglutinin (HA) and neuraminidase (NA).^[^ ^8^ ^]^ IAVs are categorized into various subtypes based on their surface HA (H1–H18) and NA (N1–N11) proteins.^[^ ^9^ ^]^ Additionally, IBVs are categorized into two lineages, *B/Victoria* and *B/Yamagata*.^[^ ^6^ ^]^ These different subtypes of influenza virus make the development of a broad‐spectrum vaccine challenging. The use of antiviral drugs, such as NA protein‐targeted oseltamivir and zanamivir, is also used to treat influenza infections.^[^ ^10^ ^]^ However, drug‐resistant strains have emerged in recent years,^[^ ^11^ ^]^ threatening public health worldwide. Lipid envelopes are conserved across influenza virus species to maintain viral integrity and infectivity.^[^ ^12^ ^]^ Therefore, a broad‐spectrum, efficient, and biocompatible antiviral strategy focusing on destroying the lipid envelope of the virus should be developed to treat various influenza viruses.

Nanotechnology has recently been widely studied for antimicrobial applications.^[^ ^13^ ^]^ For instance, copper and silver nanoparticles have been demonstrated to be potent microbicides against bacteria and viruses; however, their potential toxicity to humans and environmental health severely limits their application.^[^ ^14^ ^]^ HA and NA protein‐targeted degradation strategies using nanotechnology, such as nanosized copper (I) iodide particles, can effectively inactivate H1N1 influenza virus.^[^ ^15^ ^]^ However, they may not be effective against other subtypes of influenza *A/B* viruses owing to the variability of HA/NA proteins. To design a broad‐spectrum influenza treatment using nanotechnology, biocompatibility and effective antiviral activity must be considered. Nanozymes, discovered in 2007, are nanomaterials with enzyme‐mimetic activities that can catalyze reactions similar to those catalyzed by natural enzymes.^[^ ^16^ ^]^ Recently, nanozymes have been rapidly developed because of their easy synthesis, tunable catalytic activity, high stability, low cost, and ease of treatment.^[^ ^17^ ^]^ Our previous studies demonstrated that iron oxide nanozymes (Fe_3_O_4_, IONzymes), approved by the US Food and Drug Administration (FDA) for biomedical applications,^[^ ^18^ ^]^ possess strong peroxidase‐like activity and efficiently eliminate bacterial biofilms. This suggests that it can be used for antimicrobial applications.^[^ ^19^ ^]^ However, the antibacterial activities of these nanozymes often rely on the presence of hydrogen peroxide (H_2_O_2_), which reduces their biocompatibility and limits their application for antibacterial therapy in vivo.^[^ ^20^ ^]^

Medicinal garlic products containing organosulfur compounds, have been used to prevent and treat microbial diseases. Based on this, a novel iron sulfide nanozyme was synthesized by converting organosulfur compounds into nanoscale inorganic sulfides,^[^ ^21^ ^]^ which is safe for use and does not affect the reproductive capability.^[^ ^22^ ^]^ Moreover, iron sulfide nanozyme has a strong antibacterial activity as it disrupts pathogenic bacterial biofilms on human teeth and accelerates infected‐wound healing.^[^ ^21^ ^]^ Another metastable iron sulfides, used in an externally applied vaginal suppository to treat bacterial vaginosis efficiently counteracted resistant *G. vaginalis*.^[^ ^20^ ^]^ Further studies revealed that ferrous iron released from iron sulfides induces ferroptosis‐like death in bacteria through iron enrichment, triggering lipid peroxidation and glutathione (GSH) depletion.^[^ ^23^ ^]^ Moreover, the released polysulfide species selectively enter the bacteria and interrupt their energy metabolism by inhibiting glycolysis.^[^ ^20^ ^]^ The above antibacterial mechanisms of iron sulfides affect the basic cellular structure and functions of bacteria. Although bacteria possess a rigid cell wall comprised of peptidoglycan or lipopolysaccharide, they are comparatively simple and unicellular organisms.^[^ ^24^ ^]^ However, the influenza virus is completely different from bacteria in terms of both structure and reproduction patterns. The influenza virus is an assembly of different types of molecules consisting of genetic material (single‐stranded RNA), viral proteins, and a lipid envelope. At the biological level the main difference is that bacteria are free‐living cells that can live without relying on host cells, whereas viruses rely on the host cellular machinery to replicate, and are thus considered non‐living molecules outside the host cell.^[^ ^25^ ^]^ Moreover, viruses have been shown to induce large‐scale changes in host cellular metabolism^[^ ^26^ ^]^ and have adopted several strategies to harness cellular metabolism in accordance with their specific demands such as replication,^[^ ^27^ ^]^ which implies that targeting virus‐induced metformin provides new hope in the development of broad‐spectrum antiviral drugs including COVID‐19 treatment.^[^ ^28^ ^]^ Therefore, whether metastable iron sulfides possess broad‐spectrum antiviral activity against extracellular and intracellular virions remain unclear. Moreover, it is not known whether the antimicrobial mechanism of viruses differs from that of bacteria.

Based on the above, we recently developed IONzyme as a novel antiviral agent that acts through H_2_O_2_‐independent targeted enzymatic destruction of the conserved lipid envelope of the influenza virus.^[^ ^29^ ^]^ The novel IONzyme showed broad‐spectrum antiviral activity against H1‐H12 subtypes of influenza viruses. However, the IONzyme inactivates only extracellular viral particles and cannot enter the host cells to inhibit intracellular viral replication, which is the key stage for large‐scale production of the influenza virus. Therefore, novel antiviral agents should be biocompatible, inactivate extracellular viruses, and block intracellular viral replication.

**Scheme 1:**
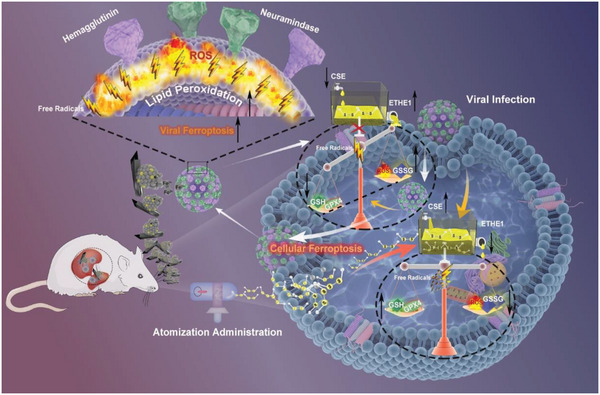
Schematic of mFeS inducing the viral ferroptosis and suppressing cellular ferroptosis against influenza virus. mFeS induced the viral ferroptosis by enhancing the lipid peroxidation level (MDA level and lipid ROS) of the viral envelope through Fe^2+^ enrichment, which further produced free radicals (•OH) to destruct HA and NA protein, resulting in a failed viral infection. In addition, Dc(mFeS), especially the released polysulfide, suppressed the cellular ferroptosis‐dependent intracellular replication of influenza virus via restoring influenza virus‐induced imbalance of sulfur metabolism (CSE, ETHE1), and the supply of intracellular S^0^. Comprehensively, mFeS&Dc through aerosol administration showed a potential preventive therapy against H1N1 virus infection in mice.

Here, we presented successful broad‐spectrum antiviral metastable iron sulfides (mFeS) against influenza virus. Surprisingly, mFeS not only induced ferroptosis‐like death of extracellular virions, but also its decoction inhibited intracellular viral replication by suppressing host cellular ferroptosis by targeting influenza virus‐hijacked cellular sulfur metabolism (**Scheme** **1**).

## 2 Results

### 2.1 mFeS Induced the Viral Ferroptosis Against Extracellular Influenza Virus

Based on a hydrothermal synthesis method, mFeS was synthesized using cysteine as a sulfur precursor.^[^ ^21^ ^]^ As shown in Figure S1, Supporting Information, mFeS exhibited nanospheres with sheet‐like growth and hexagonal nanostructures (Figure S1A, Supporting Information), whereas IONzyme displayed only nanospheres (Figure S1B, Supporting Information). Using energy dispersive spectrometry (EDS), iron and sulfide elements were found to be the components of mFeS (Figure S2, Supporting Information). The high‐resolution X‐ray photoelectron spectra (XPS) of Fe 2p and S 2p in mFeS demonstrated the presence of Fe and S (Figure S3A, Supporting Information). The peaks observed at 722.6, 709.6, and 706.5 eV were assigned to the spin‐orbit doublet of the 2p orbital of Fe^2+^ in mFeS. The two peaks at 728.4 and 712.1 eV corresponded to Fe^3+^, which indicated that the as‐prepared mFeS possesses mixed‐valence character of Fe^2+^ and Fe^3+^. Meanwhile, Figure S3B, Supporting Information, displays the S 2p spectrum, in which the peaks at 161.2 and 159.9 eV were assigned to S 2p_1/2_ and S 2p_3/2_, respectively.^[^ ^30^ ^]^ To further determine the formation of mFeS, an X‐ray diffraction (XRD) was performed (Figure S4, Supporting Information). The diffraction peak showed the coincidence of Fe_3_S_4_ and Fe_1−_ *_x_* S; the diffraction peaks occurring at 29.96°, 36.342°, and 52.357° corresponded to Fe_3_S_4_ (311), (400), and (440), respectively, and the diffraction peaks occurring at 43.65°, 36.76°, and 53.07° corresponded to Fe_1−_ *_x_* S (20‐12), (206), and (220), respectively.^[^ ^21^, ^23^ ^]^ The zeta potential data indicated that mFeS showed positive charges, implying that it was possible to adsorb the virus (negative charges) through electrostatic attraction (Figure S5, Supporting Information). Importantly, the catalytic kinetics followed typical Michaelis–Menten kinetics. mFeS exhibited not only peroxidase‐like activities in H_2_O_2_ and TMB systems (Table S1 and Figure S6, Supporting Information), but also oxidase‐like activities by oxidizing TMB (Table S1 and Figure S7, Supporting Information).

**Figure 1:**
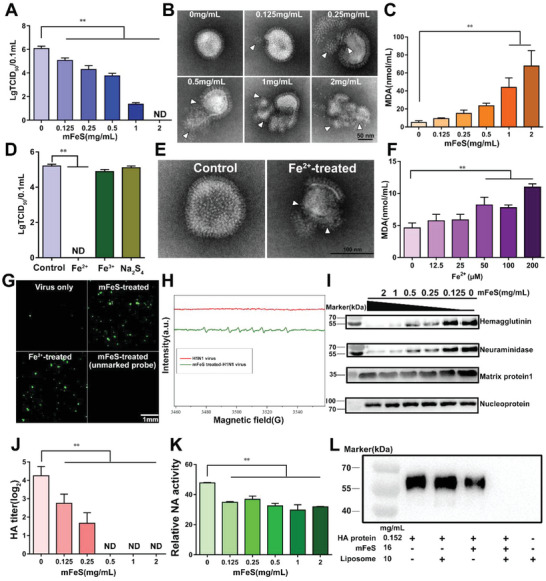
mFeS induced the viral ferroptosis. A) TCID_50_ titer of H1N1 virus following treatment with variable concentrations of mFeS. B) TEM images of H1N1 virus treated with variable concentrations of mFeS. The damages to the lipid envelope are shown with white arrows. Scale bar: 50 nm. C) The MDA levels of H1N1 virus treated with variable concentrations of mFeS. D) TCID_50_ titer of H1N1 virus following treatment with 200 µm Fe^2+^, Fe^3+^, or Na_2_S_4_, respectively. E) TEM image of the H1N1 virus after treatment with 200 µm Fe^2+^. Control: untreated H1N1 virus. The damages to the lipid envelope are shown with white arrows. Scale bar: 100 nm. F) The MDA levels of H1N1 virus treated with variable concentrations of Fe^2+^. G) BODIPY581/591‐C11 probe was used to detect the lipid peroxidation of mFeS or Fe^2+^‐treated H1N1 virus (green). Scale bar: 1 mm. H) EPR analysis of mFeS‐treated H1N1 virus. I) Western blot of HA, NA, M1, and NP proteins of H1N1 virus after treatment with mFeS. J) The HA titer and K) NA activity of mFeS‐treated H1N1 virus after 2 h. L) Western blot analysis of the degradation of HA protein in presence of liposomes after incubation with mFeS. Data shown represent the means ± SD from one of three independent experiments. All experiments were repeated in triplicate with a representative image shown. One‐way ANOVA analysis of variance with the nonparametric test is employed. * p < 0.05; ** p < 0.01. *ND*, no detection.

Next, we evaluated the potential antiviral activity of mFeS against H1N1 influenza virus (A/PR/34/8, PR8). As shown in **Figure** **1**A, mFeS showed good antiviral activity and decreased the viral 50% tissue culture infectious dose (TCID_50_) titers in a dose‐dependent manner. Notably, the titer decreased sharply from 6.10 ± 0.17 to 0.00 ± 0.00 Lg TCID_50_/0.1 mL when treated with less than 2 mg mL^−1^ of mFeS, suggesting that the virus was completely inactivated. Transmission electron microscopy (TEM) revealed that mFeS damaged the structure of the viral lipid envelope, resulting in the leakage of the internal contents of the virions (Figure 1B). To verify whether mFeS inactivated multiple subtypes of the influenza virus, the H1–H11 IAV subtypes and the IBV Victoria lineage were treated with mFeS, and the viral HA and TCID_50_ titers were evaluated. Although these subtypes were genetically distant based on the phylogenetic analysis of HA genes (Figure S8A, Supporting Information), mFeS efficiently reduced the HA and TCID_50_ titers of these subtypes in a dose‐dependent manner (Figure S8B–M, Supporting Information). Notably, when treated with >1 mg mL^−1^ mFeS, H3N2, H4N6, H5N2, H8N4, and H9N2 subtype IAVs were completely inactivated (Figure S8D,E,F,I, J, Supporting Information). Collectively, these results demonstrated that mFeS possessed broad‐spectrum antiviral activity against various subtypes of influenza viruses. Moreover, mFeS inactivated viruses with a typical lipid envelope [Newcastle disease virus (NDV), a representative enveloped virus] but not viruses without lipid envelopes [porcine circovirus type 2 (PCV‐2), a representative non‐enveloped virus] (Figures S9 and S10, Supporting Information). This finding implied that mFeS preferentially acted on enveloped viruses.

Furthermore, mFeS had the ability to induce viral lipid peroxidation by increasing the malondialdehyde (MDA) levels by 7‐fold in liposomes (Figure S11, Supporting Information). Similarly, mFeS induced strong lipid peroxidation by 2.5–10‐fold in the influenza virus in a dose‐dependent manner (Figure 1C). Moreover, natural lipoxidase, but not horseradish peroxidase (HRP) or catalase, decreased the HA titer of the influenza virus (Figure S12, Supporting Information), implying that mFeS with peroxidase‐like activity could catalyze lipid peroxidation of the influenza virus.

Our data (Figure S3, Supporting Information) and a previous study,^[^ ^23^ ^]^ confirmed that mFeS mainly consists of Fe and S. Specifically, Fe was present predominantly in its Fe^2+^ state, and only a small proportion consisted of Fe^3+^, whereas S was in the form of polysulfide. Therefore, we investigated whether Fe or S possessed the antiviral activity. Our results showed that 200 µm Fe^2+^, rather than Fe^3+^ and Na_2_S_4_, nullified both the TCID_50_ and HA titers of the influenza virus within 2 h, indicating that Fe^2+^ contributed to the antiviral activity of mFeS (Figure 1D; Figures S13–S15, Supporting Information). Furthermore, TEM observations showed serious damage to the viral lipid envelope by Fe^2+^ (200 µm) (Figure 1E). The MDA levels of the Fe^2+^‐treated H1N1 virus increased in a dose‐dependent manner by up to 2‐fold (Figure 1F), which was not observed in the Fe^3+^ and Na_2_S_4_‐treated H1N1 virus (Figure S16, Supporting Information). BODIPY581/591‐C11 is a fluorescent probe used to index lipid peroxidation and antioxidant efficacy in model membrane systems and living cells.^[^ ^23^ ^]^ We found that both mFeS and Fe^2+^ induced strong oxidant effects as shown by green fluorescence (oxidized forms) (Figure 1G). These data indicated that Fe^2+^ induced strong lipid peroxidation in the H1N1 virus, destroying the viral lipid envelope with a liposome structure. Iron dependence and lipid peroxidation are the most important signs of ferroptosis, which depends heavily on iron‐mediated free radical formation and accumulation.^[^ ^31^ ^]^ Electron paramagnetic resonance (EPR) spectrometry revealed that hydroxyl radicals (•OH) were generated by the mFeS‐treated H1N1 virus (Figure 1H; Figure S17, Supporting Information). In fact, Fenton reaction is the chemical basis of ferroptosis.^[^ ^32^ ^]^ mFeS possesses the ability to catalyze a strong Fenton reaction using Fe^2+^ to overproduce •OH free radicals, resulting in strong lipid peroxidation in the influenza virus; therefore, we termed it viral ferroptosis.

Subsequently, we examined the proteins around the viral lipid envelope by Western blotting, including two surface glycoproteins, HA and NA, which possess a transmembrane domain inserted into the lipid envelope, and matrix protein 1 (M1), which attaches to the base of the lipid envelope,^[^ ^33^ ^]^ by western blotting. As shown in Figure 1I, HA and NA proteins were degraded in a dose‐dependent manner by mFeS, whereas M1 and nucleocapsid protein (NP) appeared to be unaffected, implying that HA and NA proteins were susceptible to mFeS because of their close crosstalk with the lipid envelope. Moreover, the capacity of the HA protein to agglutinate chicken red blood cells (cRBCs) (Figure 1J; Figures S18 and S19, Supporting Information) and the NA enzymatic activity (Figure 1K) decreased, suggesting that their functions were impaired by mFeS. To verify how mFeS destroyed viral proteins, liposomes and purified HA proteins were used to simulate the viral structure. Western blotting showed that the HA protein was destroyed completely in the presence of a mFeS‐liposome system rather than by free mFeS (Figure 1L), indicating that the lipid structure was required for the destruction of the HA protein.

### 2.2 mFeS Impaired the Viral Infectibility of Influenza Virus In Vitro

**Figure 2:**
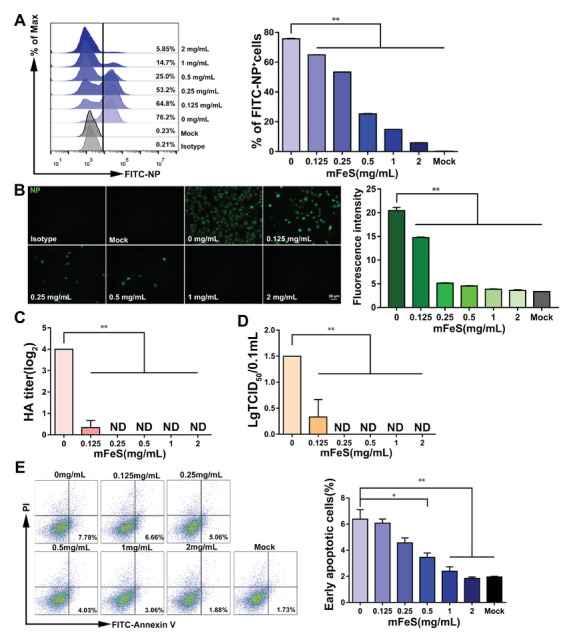
The viral infectibility of mFeS‐treated H1N1 virus in vitro. A) Percentage of FITC‐NP^+^ cells was detected as the attachment to MDCK cells by different concertation mFeS‐treated H1N1 viruses. B–E) MDCK cells were infected with mFeS‐treated H1N1 virus at an MOI of 1. B) At 24 h.p.i., MDCK cells were fixed to detect the viral intracellular replication by detecting NP protein by immunofluorescence stain (green). Fluorescence intensity was measured by ImageJ software v1.8. Scale bar: 50 µm. C,D) At 24 h.p.i., the viral release was determined by detecting HA and TCID_50_ titer. E) The proportions of early apoptotic cells (FITC‐Annexin V^+^ and PI^−^) were analyzed using flow cytometry. Data shown represent the means ± SD from one of three independent experiments. One‐way ANOVA analysis of variance with the nonparametric test is employed. * p < 0.05; ** p < 0.01. *ND*, no detection.

HA and NA proteins are responsible for the invasion and release of influenza viruses, respectively.^[^ ^34^ ^]^ The levels of attachment, intracellular replication, and release of the H1N1 virus were examined in vitro. As the NP protein was unaffected by mFeS (Figure 1I), it was used as a marker for viral quantification in the attachment assay. As shown in **Figure** **2**A and Figure S31A, Supporting Information, the percentage of FITC‐NP^+^ cells infected with mFeS‐treated virus decreased significantly in a dose‐dependent manner (*p <* 0.01). Notably, when the virus was treated with 2 mg mL^−1^ mFeS, the NP level decreased 12‐fold compared to that in the control group. In addition, a similar decrease in intracellular replication was observed in both Madin–Darby canine kidneys (MDCK) (Figure 2B) and chicken embryonic fibroblasts (CEF) (Figure S20, Supporting Information) cell infection models. During the viral release stage, cell supernatants were collected to measure HA and TCID_50_ titers. Notably, they were both undetectable when the virus was treated with mFeS at concentrations ranging from 0.25 to 2 mg mL^−1^ (Figure 2C,D), suggesting that the production of progeny virions was impaired possibly.

Furthermore, the virulence of the mFeS‐treated H1N1 virus was evaluated. The H1N1 virus can induce cellular apoptosis.^[^ ^29^ ^]^ In the group whereby the H1N1 virus was treated with 2 mg mL^−1^ mFeS, the percentage of early apoptotic cells was 1.84 ± 0.15%, similar to the mock group (1.95 ± 0.07%). However, the percentage of early apoptotic cells in the group with untreated virus was 6.38 ± 1.25%, showing a marked increase compared with the mock group (Figure 2E; Figure S31B, Supporting Information).

### 2.3 mFeS Impaired the Pathogenicity of Influenza Virus In Vivo

**Figure 3:**
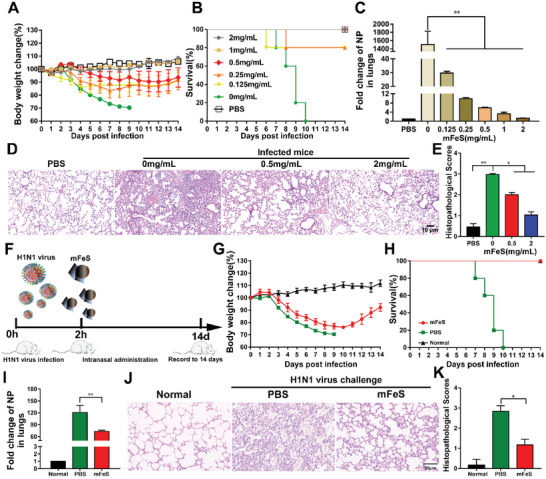
mFeS impaired the pathogenicity of influenza virus in vivo. A–E) 6‐week‐old SPF BALB/c mice were infected intranasally with mFeS‐treated H1N1 virus. A) Body weight changes. B) Mortality expressed as percent survival. Mice who lost more than 25% of their body weight were euthanized according to guidelines (*n* = 5). C) Lungs of mFeS‐treated H1N1 virus‐infected mice (*n* = 3) were collected at 3 d.p.i. to detect the level of NP gene. D,E) Representative histopathological changes and histopathologic scores in H&E‐stained lung tissues at 7 d.p.i. (*n* = 3). F) The application of Cye‐nFeS in prevention of influenza virus infection in the early stage. G,K) BALB/c mice infected by H1N1 virus (intranasal dose 10^6^ EID_50_/50 µL) were treated with 10 µL mFeS (10 mg mL^−1^) through intranasal administration 2 h after infection. G) Body weight changes. H) Mortality expressed as percent survival. Mice who lost more than 25% of their body weight were euthanized according to guidelines (*n* = 5). I) Lungs of mFeS‐treated H1N1 virus‐infected mice (*n* = 3) were collected at 7 d.p.i. to detect the level of NP gene. J,K) Representative histopathological changes and histopathologic scores in H&E‐stained lung tissues at 7 d.p.i. Data shown represent the means ± SD from one of three independent experiments. One‐way ANOVA analysis of variance with the nonparametric test is employed. * p < 0.05; ** p < 0.01. *ND*, no detection.

The pathogenicity of the mFeS‐treated H1N1 virus was evaluated using a BALB/c mouse infection model. H1N1 virus (10^6^ EID_50_/50 µL)‐challenged mice showed a 30% body weight decrease and 100% death within 10 days post‐infection (d.p.i.). However, in the mFeS‐treated H1N1 infected mice group, the body weight recovered and the survival rate increased to 80%–100% (**Figure** **3**A,B). The viral load also decreased by 50–750‐fold compared with the untreated‐mice infected group at 3 d.p.i., based on the NP gene detection (Figure 3C). Moreover, pathological changes in the lungs were examined using hematoxylin and eosin (H&E) staining, and we found that infection with intact H1N1 viruses induced severe lung injury, including alveolar wall edema thickening, inflammatory cell infiltration, and hemorrhage at 7 d.p.i. However, the histopathological score decreased with increasing mFeS concentration, implying that the group infected with the mFeS‐treated H1N1 viruses showed slight pathological injury (Figure 3D,E). These results demonstrated that mFeS could impair the pathogenicity of the influenza virus in vivo. Next, we explored whether mFeS could block viral infection at the early stage in vivo. Following infection with the H1N1 virus for 2 h, BALB/c mice were intranasally administered mFeS (Figure 3F). The mFeS‐treated mice gradually recovered after experiencing a period of weight loss and showed a 100% survival rate compared to 0% survival in the control group (Figure 3G,H). In addition, in mFeS‐treated mice at 7 d.p.i., the viral load was significantly down‐regulated (*p <* 0.01, Figure 3I), and the lung lesions healed (Figure 3J,K). Above results indicated that mFeS not only decreased the pathogenicity of the influenza virus in vivo, but also possessed therapeutic activity in the early stage of viral infection.

### 2.4 The Decoction of mFeS Suppressed Cellular Ferroptosis Against Intracellular Influenza Virus

**Figure 4:**
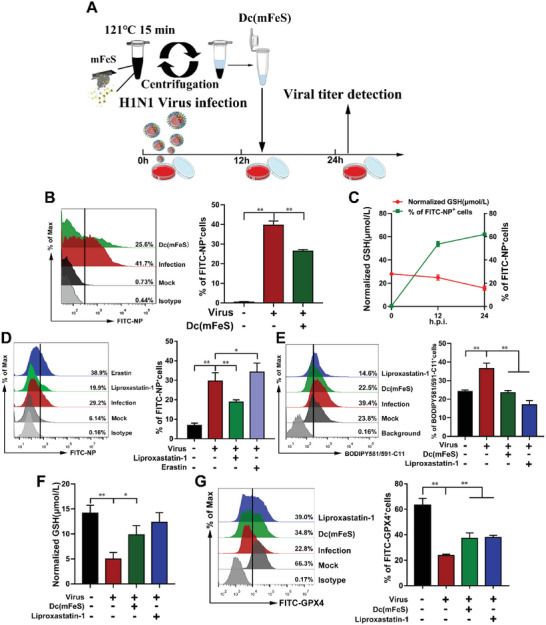
Dc(mFeS) suppressed cellular ferroptosis in influenza virus‐infected cells. A) Experimental design for Dc(mFeS) after H1N1 infection in vitro. mFeS was mixed in distilled water and autoclaved (121 °C, 15 min). Following centrifugation, Dc(mFeS) was collected and added to the H1N1 virus‐infected MDCK cells at 12 h.p.i. B) Viral load was measured at 24 h.p.i. C) MDCK cells were infected with H1N1 virus at an MOI of 1. At 12 and 24 h.p.i., the cells were collected to detect the normalized GSH levels and the percent of NP^+^ cells by flow cytometry. D) At 12 h.p.i., ferroptosis inhibitor (Liproxastatin‐1, 2 µm) or inducer (Erastin, 5 µm) was added and the percent of NP^+^ cells was detected at 24 h.p.i. by flow cytometry. E–G) MDCK cells were treated with Dc(mFeS) (4 mg mL^−1^, 500 µL) or ferroptosis inhibitor (Liproxastatin‐1, 2 µm) at 12 h.p.i., and then the cells were collected at 24 h.p.i. E) BODIPY581/591‐C11 was used as the probe to detect the lipid peroxidation by flow cytometry. F) Normalized GSH. G) GPX4 expression by flow cytometry. Quantification of the flow cytometry results as shown in the panel. Data represent the means ± SD from one of three independent experiments. One‐way ANOVA analysis of variance with the nonparametric test was employed. *p < 0.05; **p < 0.01.

Next, we investigated whether mFeS exerted a therapeutic effect after H1N1 viral infection. We found that the fluorescent probe‐labeled mFeS particles (Figure S21A, Supporting Information) barely entered the host cells until 12 h (Figures S21B and S31M, Supporting Information), implying that it did not act against intracellular viruses. The decoction of mFeS (Dc(mFeS)) was added to MDCK cells to evaluate whether it could act against viruses intracellularly (**Figure** **4**A). We found that the HA titer significantly decreased from 3.0 ± 0.00 to 0.00 ± 0.00 Log_2_ after 24 hours post‐injection (h.p.i.), and the EC_50_ value of Dc(mFeS) was 0.126 mg mL^−1^ (*p* < 0.01, *p* < 0.05, Figure S22, Supporting Information). Viral NP protein expression was significantly inhibited, as determined by flow cytometry (*p* < 0.01, Figure 4B; Figure S31C, Supporting Information). These findings indicated that Dc(mFeS) had a therapeutic effect against H1N1 viral infection in vitro. Cellular ferroptosis is morphologically, biochemically, and genetically distinct process from apoptosis, necrosis, and autophagy.^[^ ^35^ ^]^ The most important characteristics of cellular ferroptosis are a decrease in intracellular GSH, an increase in lipid peroxide, and increased level of lipid reactive oxygen species (ROS).^[^ ^36^ ^]^ We found that GSH levels decreased with an increasing viral load (Figure 4C; Figures S23 and S31D, Supporting Information). Moreover, when cellular ferroptosis was inhibited by Liproxastatin‐1, a ferroptosis inhibitor,^[^ ^36^ ^]^ the viral load (NP expression) was significantly down‐regulated (*p <* 0.01); however, when cellular ferroptosis was induced by Erastin, a ferroptosis inducer,^[^ ^36^ ^]^ the viral load was significantly up‐regulated (*p <* 0.05, Figure 4D; Figure S31E, Supporting Information). These findings suggested that H1N1 viral replication depended on cellular ferroptosis. Moreover, Dc(mFeS) remarkably inhibited the cellular lipid peroxidation level (indicated by BODIPY581/591‐C11),^[^ ^23^ ^]^ restored the imbalance of GSH, and inhibited intracellular lipid ROS level following viral infection (*p <* 0.01). The upregulation of intracellular iron, which was closely related to ferroptosis,^[^ ^36^ ^]^ was also observed during the viral infection, which was inhibited by treatment with Dc(mFeS) (Figures S24B and S31O, Supporting Information). These findings were similar to those observed for Liproxastatin‐1 (Figure 4E,F; Figures S24A,B and S31F,N,O, Supporting Information). Interestingly, Dc(mFeS) and Liproxastatin‐1 had no influence on the balance of GSH in uninfected cells (Figure S25, Supporting Information). Glutathione peroxidase 4 (GPX4), which localizes to the cytoplasm, is known as a predominant ferroptosis resistance factor.^[^ ^35^ ^]^ During the viral infection, GPX4 was depleted (*p <* 0.01); however, treatment with Dc(mFeS) or Liproxastatin‐1 post‐infection significantly suppressed GPX4 depletion (*p <* 0.01, Figure 4G; Figure S31G, Supporting Information). The above results showed that similarly to ferroptosis inhibitor, Dc(mFeS) inhibited viral replication by suppressing cellular ferroptosis.

### 2.5 The S^0^ Suppressed Cellular Ferroptosis Against Intracellular Influenza Virus

**Figure 5:**
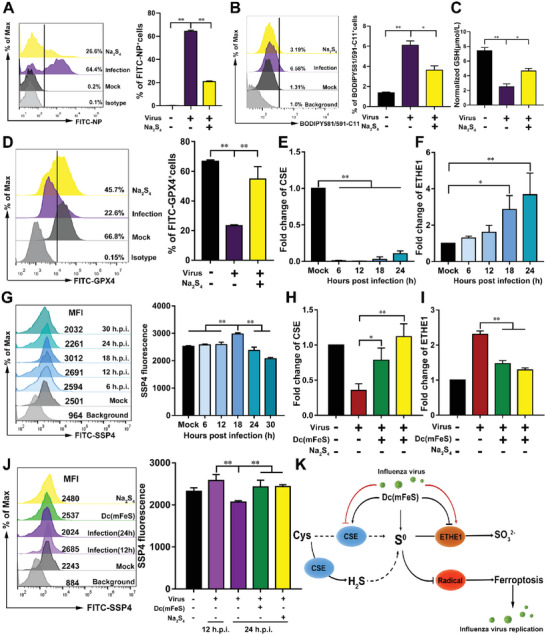
S^0^ suppressed cellular ferroptosis against intracellular influenza virus. MDCK cells were infected with H1N1 virus at an MOI of 1. A–D) After adding Na_2_S_4_ (500 µm) at 12 h.p.i., the cells were collected at 24 h.p.i. to detect the percent of A) NP^+^ cells, B) lipid peroxidation level, C) normalized GSH level, and D) GPX4 level. E,F) MDCK cells infected with H1N1 virus at an MOI of 1 were collected at different time‐points to detect the levels of ETHE1 and CSE genes by qRT‐PCR. G) S^0^ levels measured by SSP4 staining of MDCK cells. H–J) Infected MDCK cells were treated with Dc(mFeS) (4 mg mL^−1^, 500 µL) and Na_2_S_4_ (500 µm) at 12 h.p.i., and then the cells were collected to detect the levels of H) CSE and I) ETHE1 genes by qRT‐PCR, and J) the level of S^0^ measured by SSP4. K) Pathways of the supply of S^0^, elimination of intracellular radicals, and inhibition of virus infection. Quantification of the flow cytometry results as shown in the panel. Data represent the means ± SD from one of three independent experiments. One‐way ANOVA analysis of variance with the nonparametric test was employed. *p < 0.05; **p < 0.01.

To explore the mechanism by which Dc(mFeS) inhibited cellular ferroptosis, its main component (Fe^2+^)^[^ ^23^ ^]^ has been demonstrated that it did not induce lipid peroxidation at high concentrations, indicating that MDCK cells possessed high tolerance to Fe^2+^ (Figures S26 and 31P, Supporting Information). Therefore, another important component, polysulfide (Na_2_S_4_),^[^ ^23^ ^]^ was added during viral infection, and viral load and cellular ferroptosis were evaluated. Na_2_S_4_ significantly decreased the viral load based on the NP gene expression level by threefold compared with that in the infection‐only group (*p <* 0.01, **Figure** **5**A; Figure S31H, Supporting Information). Moreover, Na_2_S_4_ significantly inhibited the level of cellular lipid peroxidation (*p* < 0.01, Figure 5B; Figure S31I, Supporting Information), restored the depletion of GSH and GPX4 during viral infection (Figure 5C,D; Figure S31J, Supporting Information), and lowered intracellular lipid ROS and Fe^2+^ levels (Figures S24A,B and S31N,O, Supporting Information). Furthermore, Na_2_S_4_ suppressed cellular ferroptosis induced by Erastin in vitro (Figures S27A–D and S31Q–S, Supporting Information). Recently, the H_2_S‐generating enzyme, cystathionine *γ*‐lyase (CSE), and the persulfide‐degrading enzyme, persulfide dioxygenase (ETHE1)‐based disorders of sulfur metabolism depleted intracellular S^0^ species, including polysulfide, leading to the inhibition of free radicals scavenging, thereby, promoting cellular ferroptosis.^[^ ^37^ ^]^ As shown in Figure 5E,F, down‐regulated CSE and up‐regulated ETHE1 expression levels were observed during viral infection, along with a decrease in intracellular S^0^ level (Figure 5G; Figure S31K, Supporting Information), resulting in the accumulation of intracellular radicals, mainly phenoxyl radicals (Figure S28, Supporting Information) which could drive cellular ferroptosis.^[^ ^37^ ^]^ To restore the level of intracellular S^0^, Dc(mFeS) or Na_2_S_4_ was added to infected cells. As shown in Figure 5H–J, and Figure S31L, Supporting Information, the imbalance in CSE and ETHE1 expression levels was restored and the level of intracellular S^0^ showed a marked increase, which further decreased the level of intracellular radicals (Figure S28, Supporting Information). These results showed that Dc(mFeS) suppressed cellular ferroptosis mainly by restoring the influenza virus‐induced imbalance of CSE and ETHE1 and the supply of S^0^ to correct the reprogrammed cellular sulfur metabolism, thereby decreasing intracellular radicals and inhibiting viral replication (Figure 5K).

### 2.6 mFeS Effectively Prevented and Treated Influenza Virus Infections

**Figure 6:**
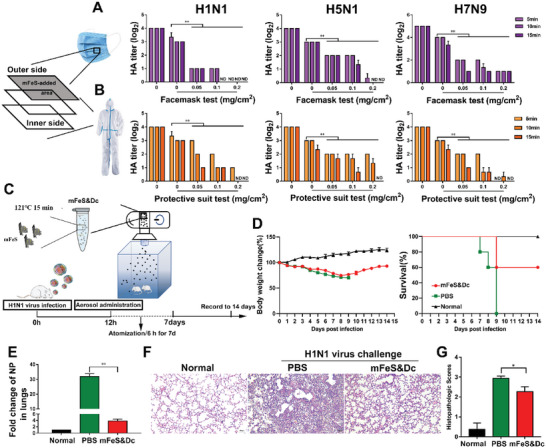
The applications of mFeS against influenza virus. A,B) Different doses of mFeS were loaded on the outer side of selected area (4 cm^2^) of facemasks or protective suits, respectively. After drying for 30 min, influenza virus was sprayed onto the outermost layer. After standing for 1 h, the population of the living virus on the antiviral facemasks and protective suits were harvested, and then HA titers of H1N1, H5N1, and H7N9 virus were detected. C) BALB/c mice were treated with mFeS&Dc (50 µg mL^−1^, 2 mL) through aerosol administration at 12 h.p.i. with a lethal dose (intranasal 10^6^ EID_50_/50 µL H1N1 virus). D) Body weight changes and mortality expressed as percent survival. Mice who lost more than 25% of their body weight were euthanized according to guidelines (*n* = 5). E) Lung tissues of treated mice (*n* = 3) were collected at 7 d.p.i. The NP expression level of lungs was measured as a marker for viral load. F,G) Representative histopathological changes and histopathologic scores in H&E‐stained lung tissues at 7 d.p.i. (*n* = 3). Data shown represent the means ± SD from one of three independent experiments. One‐way ANOVA analysis of variance with the nonparametric test is employed. * p < 0.05. ** p < 0.01. *ND*, no detection.

Personal protective equipment (PPE), such as facemasks and protective suits, is generally considered the best way to prevent influenza infections.^[^ ^38^ ^]^ mFeS can be stored for at least one and a half years and still maintained its good antiviral efficacy (Figure S29, Supporting Information). Considering the good stability of mFeS, we explored the possibility of coating mFeS particles onto facemasks and protective suits, and evaluated their antiviral effects against influenza viruses, including H1N1, H5N1, and H7N9.^[^ ^3^, ^39^ ^]^ As shown in **Figure** **6**A,B, non‐mFeS‐coated facemasks and protective suits did not effectively inactivate the virus. However, those coated with mFeS effectively inhibited the viral infection. Notably, the effect was even observed at a low concentration of mFeS of 0.2 mg cm^−2^; the HA titers (Log_2_) of the H1N1 virus quickly dropped to 0.00 ± 0.00 within 10 min. Similarly, mFeS‐loaded facemasks and protective suits provided good protection against H5N1 and H7N9 subtypes of influenza viruses within 15 min. Therefore, mFeS can be developed as an ideal antiviral PPEs to provide maximum antiviral protection against influenza virus infections.

Furthermore, we explored the potential therapeutic effect of the mFeS combined with the decoction (mFeS&Dc) against the influenza virus. Biosafety evaluation is one of the main concerns in the application of nanoparticle materials; therefore, the biosafety of mFeS&Dc was evaluated in vivo and in vitro. We did not observe any cytotoxic effects on MDCK, A549, 293T, or Vero cells (Figure S30A,B, Supporting Information). In vivo, mice treated with mFeS&Dc via nasal administration did not show a significant decrease in body weight within 14 days compared to the control group that did not receive mFeS&Dc (Figure S30C, Supporting Information). Moreover, severe lesions were not observed in the lungs of mice treated with mFeS&Dc for 7 days (Figure S30D, Supporting Information). In addition, the blood routine and blood biochemical indicators of mice treated with mFeS&Dc also suggested that mFeS&Dc was safe for use (Figure S30E,F, Supporting Information). Recently, atomization has been widely used for the treatment of respiratory diseases, such as influenza^[^ ^40^ ^]^ and COVID‐19 infections.^[^ ^41^ ^]^ Following infection with a lethal dose of influenza virus (10^6^ EID_50_/50 µL H1N1 virus), BALB/c mice were administered either mFeS&Dc or PBS every 6 h through aerosol atomization after 12 h.p.i. (Figure 6C). As shown in Figure 6D, the body weight of mFeS&Dc‐treated mice gradually increased after a period of weight loss and showed a 60% survival rate compared to 0% in the control group. In addition, the viral load was significantly down‐regulated (*p <* 0.01), and lung lesions were recovered in mFeS‐treated mice at 7 d.p.i. (Figure 6E–G). These results indicated that mFeS may have a potential therapeutic effect against the influenza virus.

## 3 Discussion

This study provided a prospective antiviral strategy against the influenza virus using mFeS, which showed the following features: i) mFeS inactivates the extracellular influenza virus by inducing viral ferroptosis depending on Fe^2+^. ii) mFeS possesses broad‐spectrum antiviral activity against various influenza *A/B* viruses. iii) mFeS impairs the viral infectibility and pathogenicity of influenza virus in vitro and vivo. iv) Dc(mFeS), especially the released polysulfide, suppresses host cellular ferroptosis, inhibiting the intracellular replication of the influenza virus by restoring sulfur metabolism. v) mFeS can be coated with PPEs, including facemasks and protective suits, to effectively prevent influenza virus infection. Moreover, the administration of mFeS&Dc through aerosol atomization can provide an effective treatment for influenza virus‐infected mice. Collectively, these features provide a comprehensive antiviral strategy against influenza viruses, which can be applied in biomedicine.

mFeS induces a high level of lipid peroxidation and the accumulation of lipid ROS in the viral lipid envelope through the increased level of free radicals, mainly •OH, depending on Fe^2+^, which is mediated by the Fenton reaction.^[^ ^42^ ^]^ Ferroptosis, a novel form of cell death characterized by iron dependence and lipid peroxidation, was first defined in 2012^[^ ^35^ ^]^ and is essentially based on the Fenton reaction. Although ferroptosis‐like death in bacteria was reported in our previous study,^[^ ^23^ ^]^ bacteria are analogous in structure to cells, resulting in a high similarity between bacterial ferroptosis‐like death and cellular ferroptosis. However, bacteria differ markedly from virus in terms of structure, metabolism, and life patterns. Owing to the lack of a defense mechanism to regulate lipid peroxidation, it was easy for virus to produce the Fenton reaction, which resulted in the generation of •OH free radicals. In fact, our study observed that the mFeS‐treated H1N1 virus possessed typical ferroptosis‐like hallmarks, including iron‐dependent lipid peroxidation and the accumulation of lipid ROS. Therefore, this is the first study to report viral ferroptosis. In addition, our previous study demonstrated that IONzyme with high enzymatic activity inactivated influenza virus by catalyzing lipid peroxidation.^[^ ^29^ ^]^ Therefore, we speculated that mFeS with oxidase‐like activity catalyzed the Fenton reaction to further accelerate the process of viral ferroptosis.

The influenza virus is surrounded by a lipid envelope derived from the host cell membrane,^[^ ^43^ ^]^ which forms a liposome‐like structure with HA and NA surface proteins inserted into the lipid envelope. Disintegration of the viral lipid envelope damages its conserved transmembrane counterparts of HA and NA. This mechanism provides the basis for a broad‐spectrum antiviral strategy that avoids the highly variable regions of HA and NA.^[^ ^44^ ^]^ In this study, mFeS not only induced lipid peroxidation in the viral lipid envelope with increased levels of free radicals (•OH), but also disintegrated nearby proteins, including HA and NA proteins. Lipid peroxidation affects the structure and activity of proteins.^[^ ^45^ ^]^ Moreover, the lipid envelope of influenza viruses contains polyunsaturated fatty acids, mostly phospholipids,^[^ ^46^ ^]^ which are vulnerable to free radical oxidation and can contribute to reactions that increase damage to biomolecules.^[^ ^47^ ^]^ The increased level of free radicals (•OH) caused by mFeS, possibly through the Fenton reaction, resulted in the cascaded disintegration of the viral envelope and nearby proteins, especially HA and NA proteins, implying a potential mechanism of impaired virulence of the influenza virus in vitro and in vivo.

Our study demonstrated that mFeS inactivated not only H1‐H11 subtype IAVs but also IBV (Victoria), showing broad‐spectrum antiviral activity against the influenza virus. Influenza A and B viruses all possess eight separate RNA segments that code for ten viral proteins, including HA and NA glycoproteins, which determine the main function of the influenza virus.^[^ ^9^ ^]^ However, HA and NA proteins mutated rapidly, resulting in a high level of antigenic shift.^[^ ^8^ ^]^ Notably, the viral envelope consists of a lipid bilayer conserved in all influenza A and B viruses.^[^ ^12^ ^]^ Therefore, disintegration of the viral envelope and incidental disruption of HA and NA proteins contributed to the broad antiviral properties of mFeS. Furthermore, SARS‐CoV‐2, which causes the novel COVID‐19, is a typical enveloped virus,^[^ ^48^ ^]^ suggesting that mFeS may be a promising antiviral agent against SARS‐CoV‐2. Except for the aim of extracellular broad‐spectrum anti‐influenza virus, achieving the intracellular broad‐spectrum anti‐influenza virus through the interference of cellular metabolism may be a prior choice against frequent variation virus such as influenza virus, SARS‐CoV‐2, Ebola virus, and Zika Virus.

Cellular ferroptosis is a novel, iron‐dependent, non‐cellular apoptotic pattern of death with necrotic morphology.^[^ ^35^ ^]^ A recent study indicated that NDV induced cellular ferroptosis through the accumulation of intracellular lipid ROS, depletion of intracellular GSH, and reduced expression of GPX4. These findings were further confirmed using a ferroptosis inhibitor (Liproxastatin‐1) and ferroptosis activator (Erastin).^[^ ^49^ ^]^ Previous studies have suggested that influenza virus infection can cause an excessive accumulation of intracellular ROS.^[^ ^50^ ^]^ Growing evidence indicates that replication of the influenza A virus is regulated by the redox state of the host cells, including the GSH content.^[^ ^51^ ^]^ Our study revealed a relationship between the intracellular replication of influenza virus and cellular ferroptosis. We found that influenza virus infected MDCK cells through the accumulation of lipid peroxidation products and lipid ROS, depletion of intracellular GSH, and reduced expression of GPX4, all of which met the major criteria for cellular ferroptosis. Moreover, viral intracellular replication was inhibited by Liproxastatin‐1, which demonstrated that the intracellular replication of influenza virus depended on cellular ferroptosis. Although mFeS showed efficient extracellular antiviral activity, it possessed a poor ability to enter cells to block intracellular viral replication. Inspired by the application of traditional Chinese decoction in the treatment of factures, skin wounds, and general pain, Dc(mFeS) was produced and used to kill bacteria as an antibacterial therapy in our previous study.^[^ ^23^ ^]^ Notably, this study demonstrated that Dc(mFeS) possessed the ability of inhibiting viral intracellular replication. Furthermore, a previous study demonstrated that the aqueous form of Dc(mFeS) contained ferrous iron and polysulfide, in which a high S/Fe ratio exceeded 3‐fold when the mFeS concentration was 4 mg mL^−1^.^[^ ^23^ ^]^ This is an effective concentration for antiviral therapy in vitro. Specifically, Fe^2+^ accounted for more than 93% of the total released iron, and sulfur was released in its major state as a polysulfide.^[^ ^23^ ^]^ Although ferrous iron can enter cells via divalent metal transporter uptake^[^ ^52^ ^]^ and is a key factor for intracellular bacterial death,^[^ ^23^ ^]^ the host cells showed high tolerance to ferrous iron to avoid intracellular ferroptosis. However, this was not observed for bacteria.^[^ ^53^ ^]^ It is worth noting that polysulfide, as the most important component of Dc(mFeS), can also quickly permeate the cell membrane.^[^ ^54^ ^]^ Other studies have demonstrated that sulfide has an antioxidant effect on microorganisms through the interconversion between GSH and GSSH.^[^ ^55^ ^]^ Moreover H_2_S produced by polysulfide can maintain intracellular GSH levels by decreasing the activity of GSH‐catabolizing enzymes.^[^ ^56^ ^]^ In this study, polysulfide showed an inhibitory effect on cellular ferroptosis induced by the ferroptosis inducer (Erastin). Furthermore, we observed that Dc(mFeS), especially polysulfide, inhibited viral replication by recovering the imbalance of GSH and GPX4, and inhibiting the expression of intracellular lipid ROS in host cells, which had an effect similar to that of the ferroptosis inhibitor (Liproxastatin‐1). To explain the mechanism of this phenomenon, we noted that Dc(mFeS), especially polysulfide, restored the influenza virus‐induced imbalance between CSE and ETHE1. This promoted the production of intracellular S^0^ to scavenge intracellular radicals by affecting heme peroxidase APEX2‐mediated redox reactions.^[^ ^37^ ^]^ Furthermore, it inhibited lipid peroxidation and ferroptosis. Polysulfide from Dc(mFeS) inhibited intracellular viral replication by suppressing cellular ferroptosis. Although we previously demonstrated that Fe^2+^ of the Dc(mFeS) induced ferroptosis‐like death in bacteria, host cells such as macrophages show a high susceptibility to ferroptosis,^[^ ^57^ ^]^ which was also identified in MDCK cells in this study. Notably, host cells possess a strong regulatory mechanism to control ferrous iron‐mediated ferroptosis, whereas it is not present in bacteria.^[^ ^53^ ^]^ Especially, viruses rely on the cellular metabolism of the host to ensure the energy and macromolecules required for replication. For example, RNA viruses, upregulate both glycolysis and glycogenolysis providing TCA cycle intermediates that are essential for anabolic lipogenesis.^[^ ^26^ ^]^ Therefore, interference of metabolism might be a promising treatment strategy which was applied in COVID‐19 treatment.^[^ ^28^ ^]^ Here, we proposed a new antiviral mechanism that was significantly different from that in bacteria, expanding the application of mFeS in antiviral therapy against the influenza virus, which implied the potential broad‐spectrum antiviral strategy by targeting virus‐hijacked cellular metabolism.

In recent years, facemasks and protective suits have been widely used by the general public for the prevention of influenza and the COVID‐19 pandemic.^[^ ^38^ ^]^ Most PPEs can only intercept but not kill the virus, resulting in a potential risk of the virus spreading through PPEs. In this study, PPEs with mFeS coating effectively inactivated the influenza virus on the surface of PPEs, providing an efficient protective strategy for curbing the virus spread. During the outbreak of emerging and re‐emerging epidemics, pathogen identification and preparation of specific antiviral agents or vaccines require a long development cycle.^[^ ^58^ ^]^ However, broad‐spectrum antiviral therapeutics have the advantage of rapid application, which is conducive to controlling epidemics during the early stage. Because the lower respiratory tract is usually the primary site of influenza virus infection, it is rational to deliver antivirals by atomization to maximize the drug concentration in the lungs. Aerosol atomization has been widely used to treat influenza viruses and COVID‐19.^[^ ^59^ ^]^ mFeS showed extracellular antiviral activity based on the viral ferroptosis, and its decoction, which inhibited intracellular viral replication by inhibiting cellular ferroptosis. To exploit these characteristics, mFeS&Dc was used to treat influenza via aerosol atomization. In addition, the positive charge of mFeS enabled the nanoparticles to adsorb to the extracellular viruses (negative charges) that were ready to invade the epithelial cells through electrostatic attraction (Figure S5, Supporting Information), and contributed to viral inactivation. Moreover, Dc(mFeS) also showed good biosafety in vitro and in vivo. Therefore, the multiple activities of mFeS resulted in a good therapeutic effect against influenza virus in vivo. Further research should focus on more efficient atomization equipment and administration measures to improve the protection rate.

In summary, our findings demonstrated that mFeS exhibited a unique mechanism based on the induction of both viral ferroptosis and suppression of cellular ferroptosis against both extracellular and intracellular influenza virus. These features endowed mFeS with broad‐spectrum antiviral activity against various subtypes of influenza *A/B* virus. With the challenge of rapid evolution of the influenza virus, mFeS may be a suitable future option for the prevention and treatment of influenza.

## 4 Experimental Section

### Ethics Statement

All animal experiments were approved by the Jiangsu Administrative Committee for Laboratory Animals (Permission number: SYXKSU‐2017‐0044) and complied with the guidelines for laboratory animal welfare and ethics of the Jiangsu Administrative Committee for Laboratory Animals. All experiments involving live highly pathogenic influenza viruses were performed in authorized animal biosafety level 3 (ABSL‐3) facilities at Yangzhou University.

### Materials

NaAc and cysteine were purchased from Sangon Biotech (Shanghai, China). Na_2_S_4_ and Cell Counting kit‐8 were purchased from DOJINDO (Kumamoto, Japan). FeCl_2_ and FeCl_3_ were purchased from Aladdin (Shanghai, China). Horseradish peroxidase (HRP) was purchased from Solarbio (Beijing, China). GSH and GSSG assay kits were purchased from the Jiancheng Bioengineering Institute (Nanjing, China). Catalase, RIPA buffer, neuraminidase assay kit, and bicinchoninic acid (BCA) protein assay kit were purchased from Beyotime (Shanghai, China). Enhanced chemiluminescence was purchased from Vazyme (Nanjing, China). Ferrostatin‐1 and Erastin were purchased from MedChemExpress (Shanghai, China). Liposomes were purchased from Nanoeast (Nanjing, China). The anti‐PCV‐2 pig polyclonal antiserum was purchased from VMRD (Pullman, WA, USA). FITC‐conjugated rabbit anti‐pig and goat anti‐mouse IgG antibodies were purchased from abcam (Shanghai, China). FeRhoNox‐1 (RhoNox‐1) was purchased from Goryo Chemical (Hokkaido, Japan). A/PR/8/34 H1N1 (PR8) and different subtypes of IAVs or IBV (H1N1, H2N2, H3N2, H4N6, H5N2, H6N2, H7N9, H8N4, H9N2, H10N3, H11N2, and B/Victoria, listed in Table S2, Supporting Information) were isolated or stored in the laboratory.^[^ ^29^ ^]^ All influenza A viruses were propagated in 10‐day‐old specific pathogen‐free (SPF) embryonic chicken eggs. The influenza B virus was propagated in MDCK cells. The H1N1 virus was purified using a discontinuous sucrose density gradient to remove other molecules, according to a previously described method.^[^ ^60^ ^]^ NDV (enveloped virus) and PCV‐2 (non‐enveloped virus) were isolated, purified, and stored in the laboratory. MDCK (ATCC; CCL‐34), A549 (ATCC; CCL‐185), 293T (ATCC, CRL‐3216), Vero (ATCC, CCL‐81) and PK‐15 (ATCC CCL‐33) cells were maintained in DMEM (Gibco, Carlsbad, CA, USA) supplemented with 10% fetal bovine serum (FBS) (Gibco, Carlsbad, CA, USA) at 37 °C under 5% CO_2_. CEF cells were prepared from 10‐day‐old embryos and cultured in M199 media (Gibco, Carlsbad, CA, USA) supplemented with 10% FBS at 37 °C under 5% CO_2._

### Synthesis of mFeS

mFeS was prepared according to a previously described method.^[^ ^21^ ^]^ mFeS was synthesized in a one‐step solvothermal system by combining FeCl_3_ and NaAc in ethylene glycol. Briefly, 0.82 g FeCl_3_ was dissolved in 40 mL ethylene glycol to form a clear solution. Next, 3.6 g of NaAc was added to the solution with vigorous stirring for 30 min. Finally, cysteine (0.5 g) was added to the solution and vigorously stirred for 30 min. The mixture was then transferred to a 50 mL Teflon‐lined stainless‐steel autoclave and was autoclaved at 200 °C for 12 h. After the autoclave cooled to room temperature, a black precipitate was collected. It was rinsed several times using ethanol, and then dried at 60 °C. The synthesized nanoparticles were characterized by scanning electron microscopy (SEM, S‐4800, Hitachi, Japan), transmission electron microscopy (TEM, Tecnai 12, Philips, Netherlands), X‐ray photoelectron spectroscopy (XPS, Thermo Scientific, USA), and X‐ray diffraction (XRD, Bruker AXS, Germany).

### Decoction of mFeS

Based on the previous study where a calcination or decoction of traditional Chinese medicine was carried out, the decoction of mFeS (Dc(mFeS)) in distilled water was developed in an autoclave (121 °C, 15 min).^[^ ^23^ ^]^ Following centrifugation, Dc(mFeS) was collected.

### Transmission Electron Microscopy of Influenza Virus

Influenza virus was treated with variable concentrations of mFeS or 200 µm Fe^2+^ for 2 h. Treated influenza virus was primarily‐fixed with 2.5% glutaraldehyde at 4 °C and then negatively stained with 2% uranyl acetate. The morphology of the influenza virus was captured using a Tecnai 12 transmission electron microscope (Philips, Eindhoven, Netherlands). Digital images were acquired using a blazing fast CCD camera system (Gatan, Pleasanton, CA, USA).

### Determination of the MDA Level

MDA level, a reliable marker of lipid peroxidation, was measured using thiobarbituric acid (TBA). Variable concentrations of mFeS, Fe^2+^, Fe^3+^, or Na_2_S_4_ were mixed with purified H1N1 virus for 2 h, and then the supernatant was collected after centrifugation. The level of lipid peroxidation was detected using a commercial MDA detection kit (Jiancheng Bioengineering Institute, Nanjing, China) according to the manufacturer's instructions. The MDA concentrations were calculated based on the absorbance of TBA reactive substances at 532 nm.

### Determination of the Lipid Peroxidation

The level of lipid peroxidation was assessed using a fluorescent probe of BODIPY581/591‐C11 (Sigma‐Aldrich, St Louis, MO, USA).^[^ ^23^ ^]^ A suspension of 1 × 10^6^ cells mL^−1^ and mFeS‐or Fe^2^^+^‐treated H1N1 virus was loaded with the probe at a final concentration of 2 µm. The suspension was then incubated at 37 °C for 30 min, washed by centrifugation to remove the unbound probe, and detected by flow cytometry (Beckman Coulter, Brea, CA, USA) for intracellular level of lipid peroxidation. The level of lipid peroxidation (green) induced by mFeS‐or Fe^2^^+^‐treated H1N1 viruses was observed using a fluorescent microscope (Leica, Tokyo, Japan).

### Determination of the Intracellular ROS

Intracellular ROS levels were detected using a 2ʹ,7ʹ‐dichlorofluorescin diacetate (DCFH‐DA) fluorescent probe (Beyotime, Shanghai, China).^[^ ^21^ ^]^ According to the manufacturer's instructions, intracellular DCFH can be oxidized to DCF by ROS. Briefly, after incubating with 10 µm DCFH‐DA at 37 °C for 30 min, the ROS level, determined as the fluorescence intensity of DCF, was measured by flow cytometry

### Western Blot

Variable concentrations of mFeS were mixed with the purified H1N1 virus for 2 h. In another experiment, liposomes (10 mg mL^−1^) were mixed with HA protein of H1N1 influenza virus (0.152 mg mL^−1^) (Sinobiological, Beijing, China); subsequently, mFeS (16 mg mL^−1^) was added and mixed with the mixtures at 37 °C at neutral pH for 2 h. The supernatants from the above two experiments were collected after centrifugation and lysed with RIPA buffer (Solarbio, Beijing, China) containing protease inhibitors. Protein extracts were resolved on 12% SDS‐polyacrylamide gels (Solarbio, Beijing, China), transferred to polyvinylidene fluoride membranes (Cytiva, Freiburg, Germany), blocked with PBS containing 0.05% Tween (PBST) and 5% skimmed milk powder, and probed with antibodies specific for mAb HA, mAb NA, pAb M1, and mAb NP (abcam, Shanghai, China) followed by incubation with horseradish peroxidase‐conjugated goat anti‐mouse or peroxidase‐conjugated goat anti‐rabbit antibodies (abcam, Shanghai, China). Protein bands were visualized using the enhanced chemiluminescence in an image analysis system (Tanon‐5200, Tanon, Shanghai, China).

### Hemagglutination Assay and TCID_50_ Detection

To assess the antiviral activity of mFeS against influenza virus, variable concentrations of mFeS were mixed with the influenza virus at different times. mFeS was pelleted by centrifugation, and the supernatant was collected to detect viral titers by HA assay and TCID_50_ assay.^[^ ^29^ ^]^ To measure the HA titers, the individual supernatants were serially diluted twofold from 2^−1^ to 2^−11^. A 1% cRBC suspension was added to detect the HA titer of IAV. A 1% guinea pig red blood cell suspension was added to determine the HA titer of IBV. The viral HA titers were evaluated after the plates were incubated for 10 min at 37 °C. To measure the TCID_50_ titers, the individual supernatants were serially diluted 10‐fold from 10^−1^ to 10^−9^, and each dilution (10^−5^–10^−9^) was inoculated into confluent CEF cell monolayers for IAV detection or MDCK cell monolayers for IBV detection at 37 °C for 1 h. After 1 h, the monolayer was rinsed with PBS, overlaid with medium (1% FBS in M199 or 1% FBS in MEM), and incubated at 37 °C for 72 h. The LgTCID_50_ per 0.1 mL was calculated using the Reed–Muench method as described previously.^[^ ^61^ ^]^

### Neuraminidase Activity Assay

H1N1 virus was treated with variable concentrations of mFeS for 2 h. The treated H1N1 virus was analyzed using a neuraminidase assay kit according to the manufacturer's instructions. Briefly, 10 µL of treated H1N1 virus was mixed with 70 µL detection buffer, 10 µL NA fluorogenic substrate, and 10 µL double‐distilled water. Fluorescence was measured at an emission wavelength of 450 nm and an excitation wavelength of 322 nm, and monitored using a multifunctional microplate reader (Tecan, Mannedorf, Switzerland). NA activity was shown as the intensity of fluorescence above the background value for the supernatant without the virus.

### Immunofluorescence

MDCK or CEF cells were infected with mFeS‐treated H1N1 virus at an MOI of 1 for 24 h; the cells were fixed with PBS containing 4% paraformaldehyde for 20 min, permeabilized with PBS containing 0.2% Triton X‐100 (Sigma, St. Louis, MO, USA) for 5 min, and then blocked with 5% bovine serum albumin in PBS for 1 h. Cells were then washed three times with PBS and incubated at 37 °C for 1 h with mAb NP (abcam, Shanghai, China) followed by incubation with FITC‐conjugated goat anti‐mouse IgG. After washing, the cells were observed under a fluorescence microscope.

For immunofluorescence detection of PCV‐2, PK‐15 cells were infected with mFeS‐treated or untreated PCV‐2 at an MOI of 0.5 for 24 h. After washing, fixation, saturation, and blocking, the cells were incubated at 37 °C for 1 h with using anti‐PCV‐2 pig polyclonal antiserum, followed by incubation with FITC‐conjugated rabbit anti‐pig antibody. The cells were then washed and examined under a fluorescence microscope. Cells positive for PCV‐2 viral antigens were counted in six fields of view.

### Flow Cytometry

To detect the attachment ability of the mFeS‐treated H1N1 virus (pretreated for 2 h), MDCK cells were infected with mFeS‐treated H1N1 virus at an MOI of 1. Cells without fixation and permeabilization were collected after 1 h to determine the percentage of FITC‐NP^+^ cells by flow cytometry.

MDCK cells were stained with the fluorescent mAbs, including FITC‐NP (abcam, Shanghai, China) or isotypes, and then detected by flow cytometry. To detect the level of GPX4, MDCK cells were stained with the fluorescent mAb, including PE‐GPX4 (abcam, Shanghai, China) or isotypes, and then detected by flow cytometry. Data were analyzed using FlowJo V10 software (FlowJo LLC, Ashland, OR, USA).

### Apoptosis Assay

MDCK cells were infected with mFeS‐treated or untreated H1N1 virus at an MOI of 1. At 24 h.p.i., MDCK cells were stained with fluorescein isothiocyanate (FITC)‐conjugated annexin V and propidium iodide (PI) according to the manufacturer's instructions (BD Biosciences, San Jose, CA, USA). The stained cells were analyzed by flow cytometry. Early apoptotic cells were characterized by annexin V^+^ and PI^−^ staining. The cell proportions were analyzed using the FlowJo V10 software.

### Consumption of GSH In Vitro

The levels of glutathione were measured using GSH and GSSG assay kits according to the manufacturer's instructions. Cells were collected to detect the levels of T‐GSH and GSSG at an absorbance of 405 nm using a microplate reader (Bio‐Rad, Richmond, CA, USA). Glutathione levels were calculated according to the established standard curve lines.

### S^0^ Measurements

MDCK cells were infected with the H1N1 virus at an MOI of 1 for different infection times. SSP4 (DOJINDO, Kumamoto, Japan) in PBS (10 µm final concentration) was then added to the culture wells. After 30 min of incubation, the cells were detached, and washed in PBS. Fluorescent staining was performed using flow cytometry (Ex 488 nm/Em 520 nm).

### Electron Spin Resonance Spectroscopy

Virus or cells were collected and incubated with DMPO (50 mm) before detection. An electron paramagnetic resonance (EPR) spectrometer (Bruker, Karlsruhe, German) was used to detect the level of free radicals. This work was supported by Testing Center of Yangzhou University.

### Biosafety Evaluation of mFeS

In vitro, variable concentrations of mFeS&Dc were added during the MDCK and A549 cell culture. Cellular morphology was observed using an electronic microscope post treatment. The cell counting kit‐8 assay was used to detect the cytotoxicity of mFeS&Dc in MDCK, A549, 293T and Vero cells. BALB/c mice (*n* = 5) were treated with 2 mL mFeS combined with a decoction (50 µg mL^−1^) through aerosol administration. Atomization was performed every 6 h for 7 days. Morbidity was evaluated by monitoring weight changes over a 14‐day period and plotted as a percentage of the weight of the animals on the day of inoculation (day 0). Blood and serum of treated mice were collected to detect the blood routine and blood biochemical indicators by animal hospital of Yangzhou university.

### Treatment for Influenza Virus Infection in a Murine Model

6‐week‐old female BALB/c mice were inoculated intranasally with 10^6^ EID_50_ of H1N1 virus in 50 µL PBS. The mice were divided into four groups (13 mice per group). Two groups of mice infected with H1N1 virus were treated with 2 mL mFeS&Dc (50 µg mL^−1^) or PBS through atomization 12 h after infection. Atomization was performed every 6 h for 7 days. Two other groups of mice infected with H1N1 virus were treated intranasally with 10 µL mFeS (10 mg mL^−1^) or PBS 2 h after infection. The animals were monitored daily for weight loss and mortality over a period of 14 days. Three mice from each group were euthanized at 7 d.p.i. to detect pathological changes and viral replication levels in murine lungs, as described previously.^[^ ^29^ ^]^

### Quantitative Reverse Transcription‐PCR

The NP gene of the influenza virus from the lungs of mice was detected by qRT‐PCR assess the viral load. The housekeeping gene *β*‐actin (F: CTTCTTGGGTATGGAATCCT, R: GTAATCTCCTTCTGCATCCTG) was used as the internal standard. The primer sequence used for amplification was NP (F: GGGCAGAACGTCTGACATGA, R: GGGTTCGTTGCCTTTTCGTC).

MDCK cells infected with H1N1 virus at an MOI of 1 were collected at 6, 12, 18, and 24 h.p.i. Quantitative reverse transcription‐PCR (qRT‐PCR) was used to detect the expression levels of ETHE1 and CSE. The primer sequences used for amplification were as follows: ETHE1 (F: GAGGCCGTTCTGATCGACC, R: GCAGTGGGTATTCACAGCATAG), CSE (F: CCTGGGCTGCCCTCTCATCCA, R: TGCCGGAAGCTCAGCAAGGC), and *β* ‐actin (F: AAGGACGGCCTCGGTTTC, R: GTGTTCAGATTTGTGAGTGGG).

### Application of mFeS in PPEs

Based on traditional facemasks with absorbent filter cotton (three layers), 0.2, 0.1, 0.05, and 0 mg cm^−2^ mFeS were loaded on the outer side of the selected area (4 cm^2^). For the protective suits (three layers), 0.2, 0.1, 0.05, and 0 mg cm^−2^ mFeS were loaded on the outer side of the selected area (4 cm^2^). After drying under airflow for 30 min, influenza virus was sprayed onto the outermost layer. After incubation for 1 h, both the control area (equal volume PBS) and the mFeS area were placed into a tube containing 200 µL PBS. After washing and extrusion, viral suspensions were harvested, and virus titers were measured using the HA and TCID_50_ methods.

### Statistical Analysis

Results were expressed as the mean ± SD and analyzed using GraphPad Prism 8 software (GraphPad Software Inc, San Diego, CA, USA). Statistical analyses were performed using SPSS, version 22.0 (IBM, Armonk, NY, USA). The unpaired Student's two‐sided *t*‐test was used to determine the differences between the two groups. One‐way analysis of variance (ANOVA) or two‐way ANOVA was used to determine differences between multiple factors. *p < 0.05, **P < 0.01 were deemed to be statistically significant. *ns*, no significant. *ND*, no detection. Data were combined from at least three independent experiments, unless otherwise stated.

## Conflict of Interest

The authors declare no conflict of interest.

## Author Contributions

T.Q., D.P., L.G., and X.M. conceived and designed the study. X.M., Y.Y., Y.C., and W.B. performed the experiments. T.Q., X.M., L.G., and YC.Y. analyzed the data. D.P., S.C., and X.L. provided critical resources. T.Q. and X.M. wrote the manuscript. All authors have contributed to, reviewed, and approved the manuscript.

## Supporting information

Supporting Information

## Data Availability

The data that support the findings of this study are available from the corresponding author upon reasonable request.
