# Supplementary material for: Bidirectionally Regulating Viral and Cellular Ferroptosis with Metastable Iron Sulfide Against Influenza Virus
Source: Adv Sci (Weinh). 2023 Apr 24;10(17):2206869. doi: 10.1002/advs.202206869 (PMC10265104; doi:10.1002/advs.202206869)
Supplement: Supplementary file 1 — Supporting Information [file ADVS-10-2206869-s001.pdf]

ADVANCED SCIENCE
Open Access

Supporting Information

for *Adv. Sci.*, DOI 10.1002/advs.202206869

Bidirectionally Regulating Viral and Cellular Ferroptosis with Metastable Iron Sulfide Against Influenza Virus

Xinyu Miao, Yinyan Yin, Yulian Chen, Wenhui Bi, Yuncong Yin, Sujuan Chen, Daxin Peng, Lizeng Gao*, Tao Qin* and Xiufan Liu*

Supporting Information

Bidirectionally regulating viral and cellular ferroptosis with metastable iron sulfide against influenza virus

Xinyu Miao[1,2†], Yinyan Yin[3,4,5†], Yulian Chen[1], Wenhui Bi[1], Yuncong Yin[1], Sujuan Chen[1], Daxin Peng[1,2,7,8], Lizeng Gao[6]*, Tao Qin [1,2,7,8]*, Xiufan Liu[1,2,7]*

Supporting Information

Bidirectionally regulating viral and cellular ferroptosis with metastable iron sulfide against influenza virus

***Xinyu Miao*[1,2†], *Yinyan Yin*[3,4,5†], *Yulian Chen*[1], *Wenhui Bi*[1], *Yuncong Yin*[1], *Sujuan Chen*[1], *Daxin Peng*[1,2,7,8]*, *Lizeng Gao*[6]*, *Tao Qin*[1,2,7,8]*, *Xiufan Liu*[1,2,7]**

[1]College of Veterinary Medicine, Yangzhou University, Yangzhou, Jiangsu, 225009, P. R. China.

[2]Joint International Research Laboratory of Agriculture and Agri-Product Safety, the Ministry of Education of China, Yangzhou University, Yangzhou University, Yangzhou Jiangsu, 225009, P. R. China.

[3]College of Medicine, Yangzhou University, Yangzhou, Jiangsu, 225009, P. R. China.

[4]International Research Laboratory of Prevention and Control of Important Animal Infectious Diseases and Zoonotic Diseases of Jiangsu Higher Education Institutions, Yangzhou University, Yangzhou, Jiangsu, 225009, P. R. China.

[5]Guangling College, Yangzhou University, Yangzhou, Jiangsu, 225009, P. R. China.

[6]CAS Engineering Laboratory for Nanozyme, Institute of Biophysics, Chinese Academy of Sciences, Beijing, 100101, P. R. China.

[7]Jiangsu Co-Innovation Center for the Prevention and Control of Important Animal Infectious Disease and Zoonoses, Yangzhou, Jiangsu, 225009, P. R. China.

[8]Jiangsu Research Centre of Engineering and Technology for Prevention and Control

of Poultry Disease, Yangzhou, Jiangsu, 225009, P. R. China.

†These authors contributed equally to this work

*Correspondence and requests for materials should be addressed to: pengdx@yzu.edu.cn (Daxin Peng); gaolizeng@ibp.ac.cn (Lizeng Gao); qintao@yzu.edu.cn (Tao Qin)

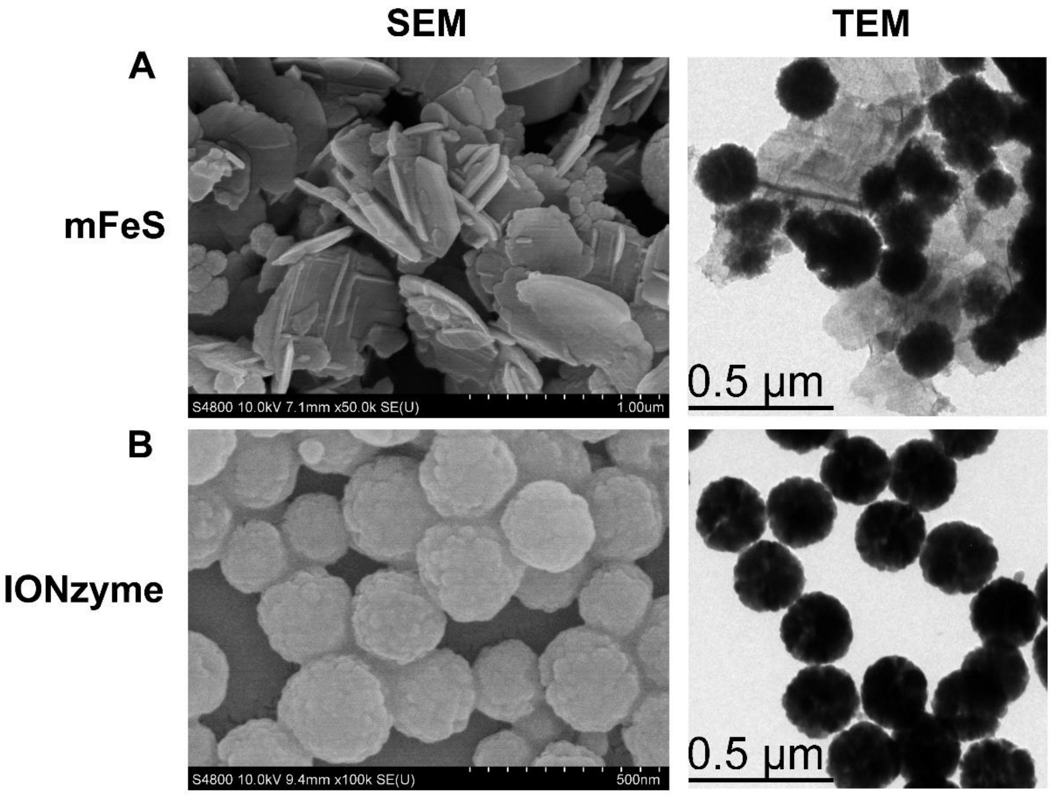

Figure S1 Characterizations of mFeS. (A) The SEM image (Scale bar: 1 μm) and TEM micrograph (Scale bar: 0.5 μm) of the prepared mFeS. (B) The SEM image (Scale bar: 0.5 μm) and TEM micrograph (Scale bar: 0.5 μm) of the prepared IONzyme. Representative images were shown.

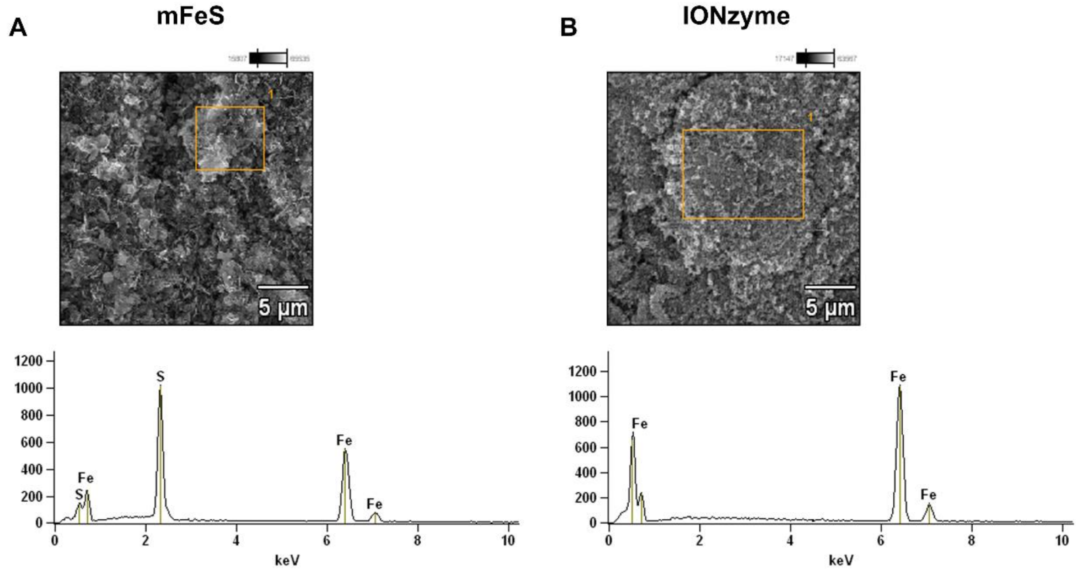

Figure S2 EDS analysis of synthesized (A) mFeS and (B) IONzyme. Scale bar: 5 μm. Representative images were shown.

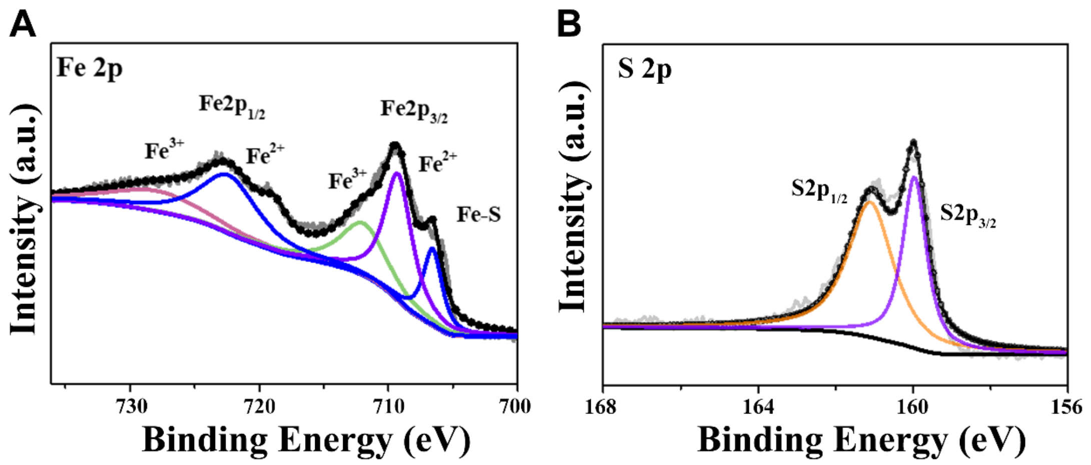

Figure S3 XPS analysis of mFeS. The corresponding deconvoluted Fe 2p (A) and S 2p spectra (B) of mFeS.

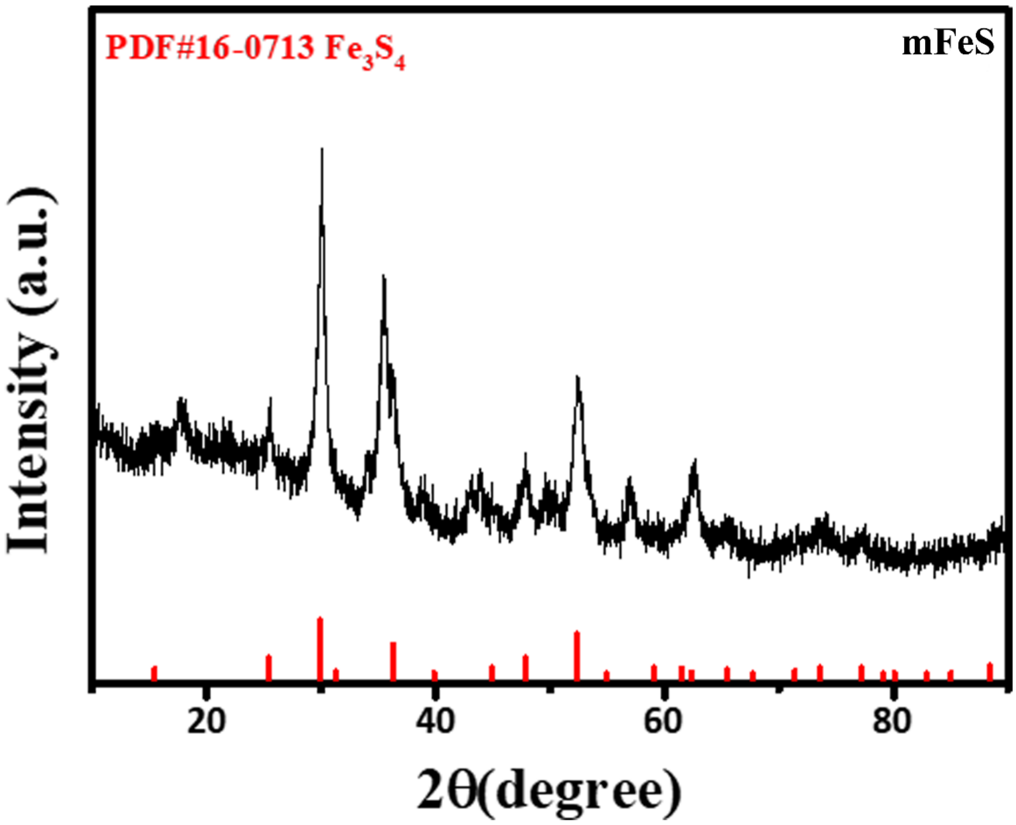

Figure S4 XRD characterization of mFeS in the two phases of Fe_3S_4 and $Fe_{1-x}S$.

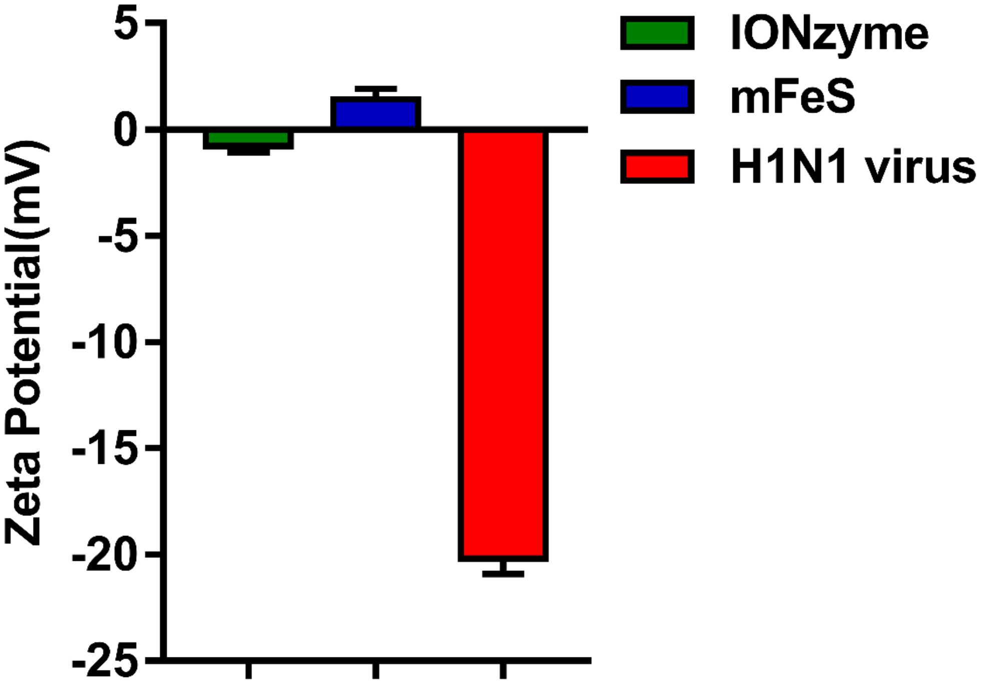

Figure S5 Zeta potential detection. Zeta potential detection of IONzyme, mFeS and H1N1 virus by Malvern Instrument Nano-ES90 (Malvern Instruments, Malvern, UK).

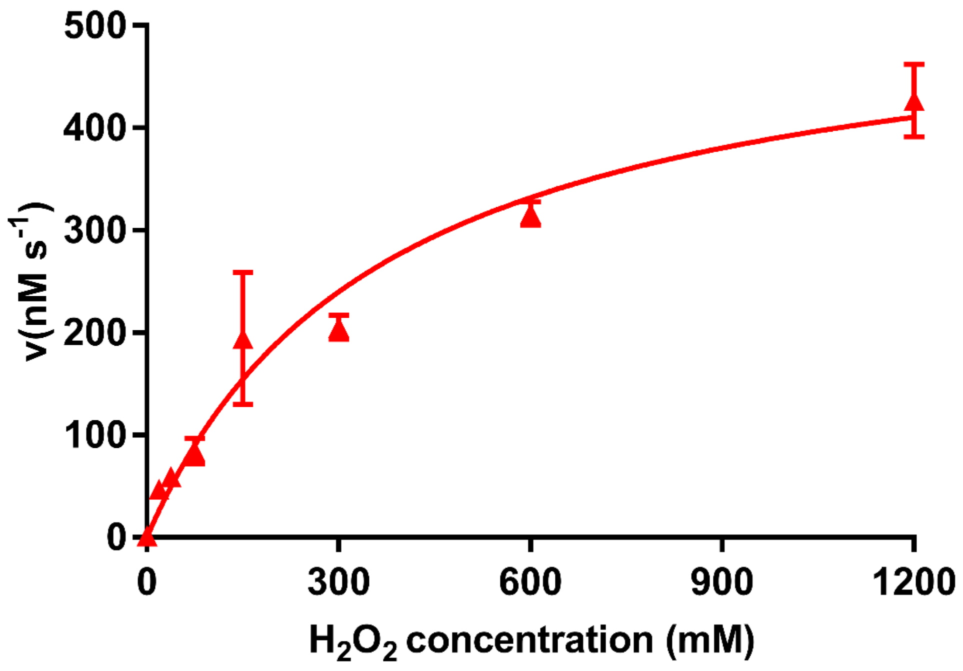

Figure S6 Michaelis-Menten kinetics for peroxidase-like activity of mFeS towards TMB and H_2O_2. The reaction was conducted in NaOAc buffer (0.1M, pH 4.5) containing 200 μL buffer solution (0.1 M NaAc, pH 4.5) in the order of 20 μL mFeS (final concentration 4 mg/mL), 4 μL of TMB (10 mg/mL), and variable concentration H_2O_2 (final concentration 0-1200 mM). Absorbance at 652 nm for TMB in the color reaction was recorded at a certain reaction time to evaluate the peroxidase-like activity. The Michaelis-Menten constant was calculated using the Lineweaver-Burk plot. The data shown are the means ± SD from one of three independent experiments.

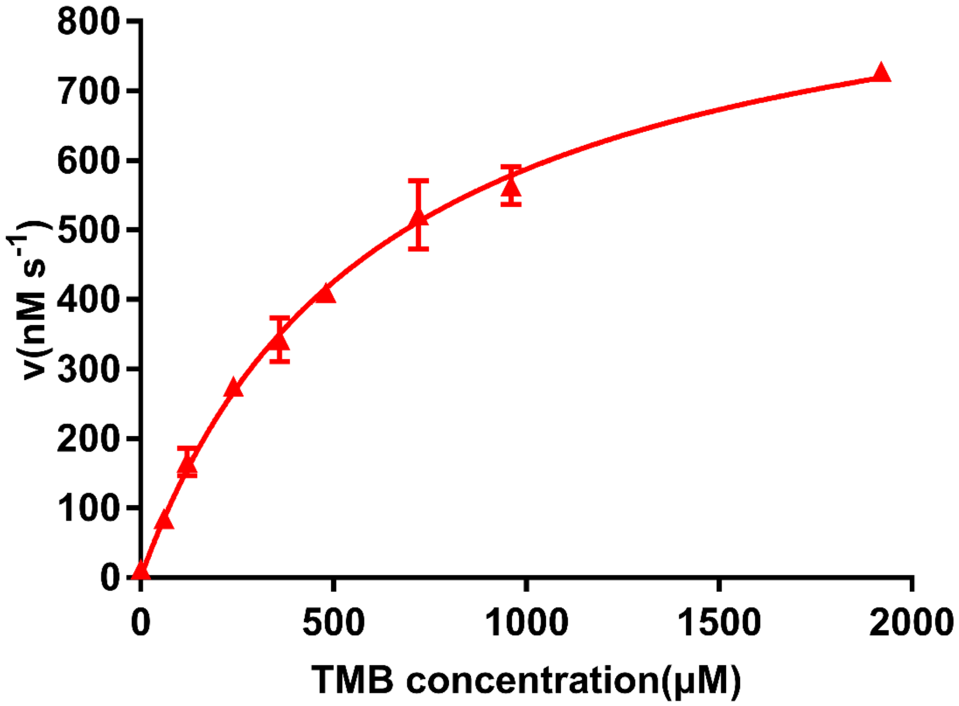

Figure S7 Michaelis-Menten kinetics for oxidase-like activity of mFeS towards TMB. The reaction was conducted in NaOAc buffer (0.1M, pH 4.5) containing 200 μL buffer solution (0.1 M NaAc, pH 4.5) in the order of 20 μL mFeS (final concentration 4 mg/mL), and variable concentration TMB (final concentration 0-2000 μM). Absorbance at 652 nm for TMB in the color reaction was recorded at a certain reaction time to evaluate the oxidase-like activity. The Michaelis-Menten constant was calculated using the Lineweaver-Burk plot. The data shown are the means ± SD from one of three independent experiments.

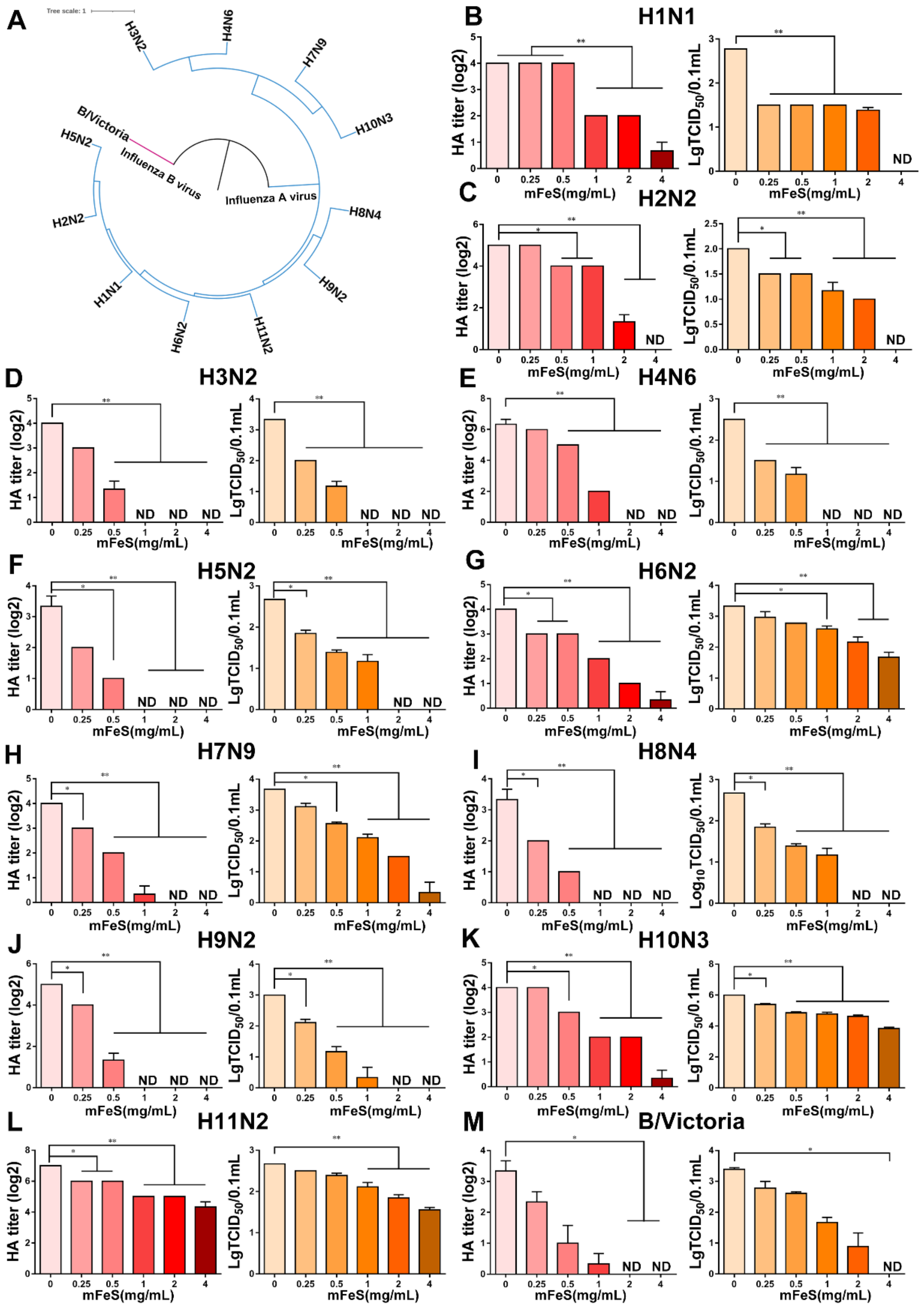

Figure S8 Broad spectrum inactivation of different subtypes of influenza virus by mFeS. (A) Phylogenetic analysis of the complete hemagglutinin genes of influenza A and B viruses. (B-M) HA and $TCID_{50}$ titers of mFeS-treated influenza viruses for 2 h, including H1 (B), H2 (C), H3 (D), H4 (E), H5 (F), H6 (G), H7 (H), H8 (I), H9 (J), H10 (K), H11 (L) and B/Victoria (M) subtypes. Data shown represent the means ± SD from

one of three independent experiments. One-way ANOVA analysis of variance with the nonparametric test is employed. * $P<0.05$; ** $P<0.01$. *ND*, no detection.

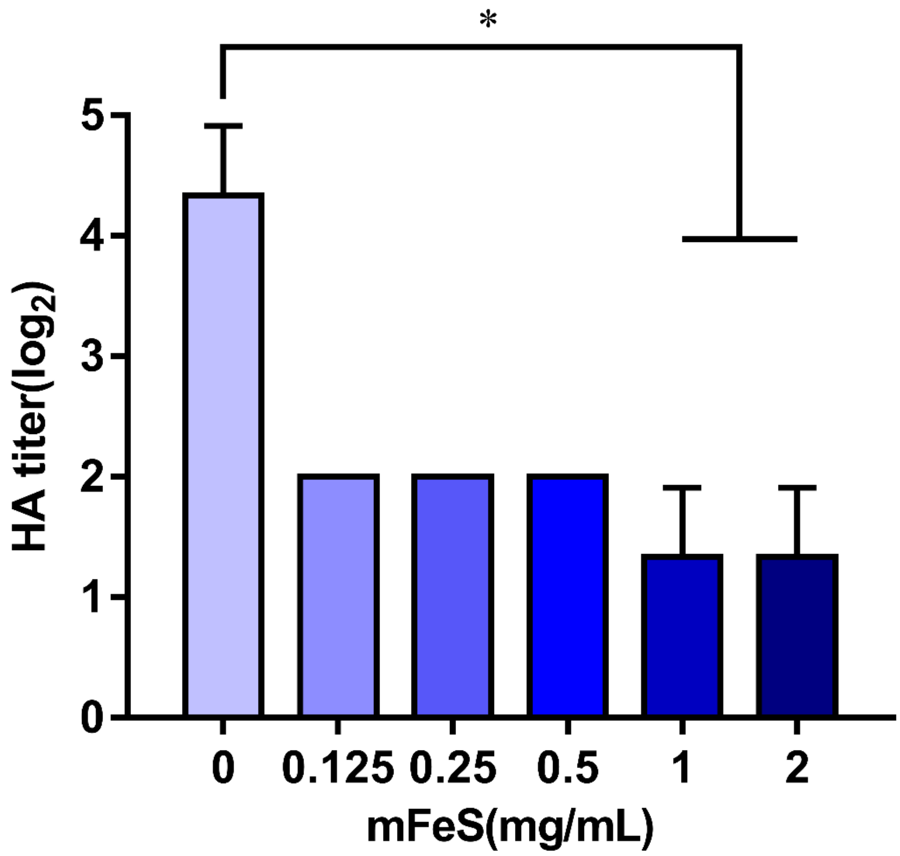

Figure S9 Antiviral activity of mFeS against the enveloped virus. NDV, a representative enveloped virus, was treated with mFeS under variable concentrations for 2 h, and the supernatant were collected to detect viral titers by hemagglutination (HA) assay. Means ± SD from one of three independent experiments is presented. One-way ANOVA analysis of variance is employed. * $p < 0.05$.

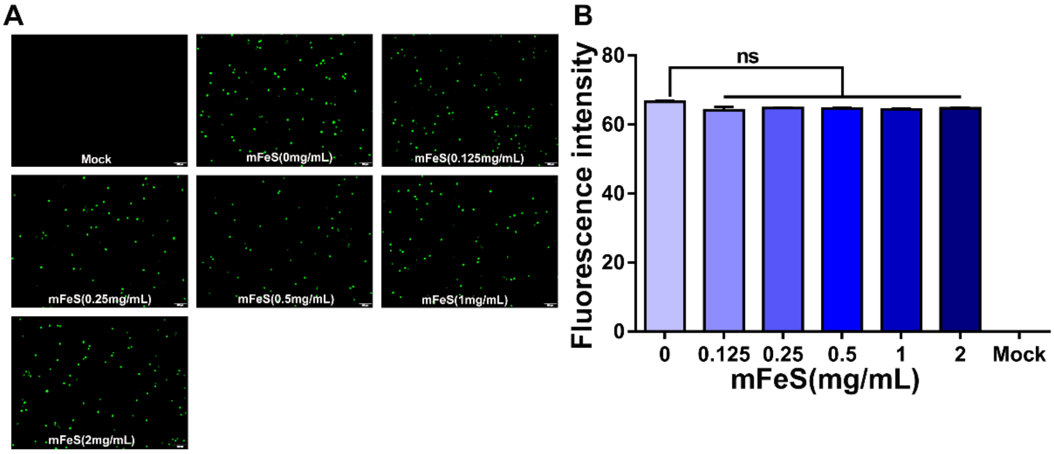

Figure S10 Antiviral activity of mFeS against the non-enveloped virus. Variable concentrations of mFeS were mixed with PCV-2 (representative non-enveloped virus) for 2 h. (A) The number of PCV-2-infected PK15 cells was detected by immunofluorescence assay. Scale bar: 500 μm. (B) Fluorescence intensity (green) was measured by imageJ software v1.8. All experiments were repeated in triplicate with a representative image shown. One-way ANOVA analysis of variance is employed. *ns,* no significant.

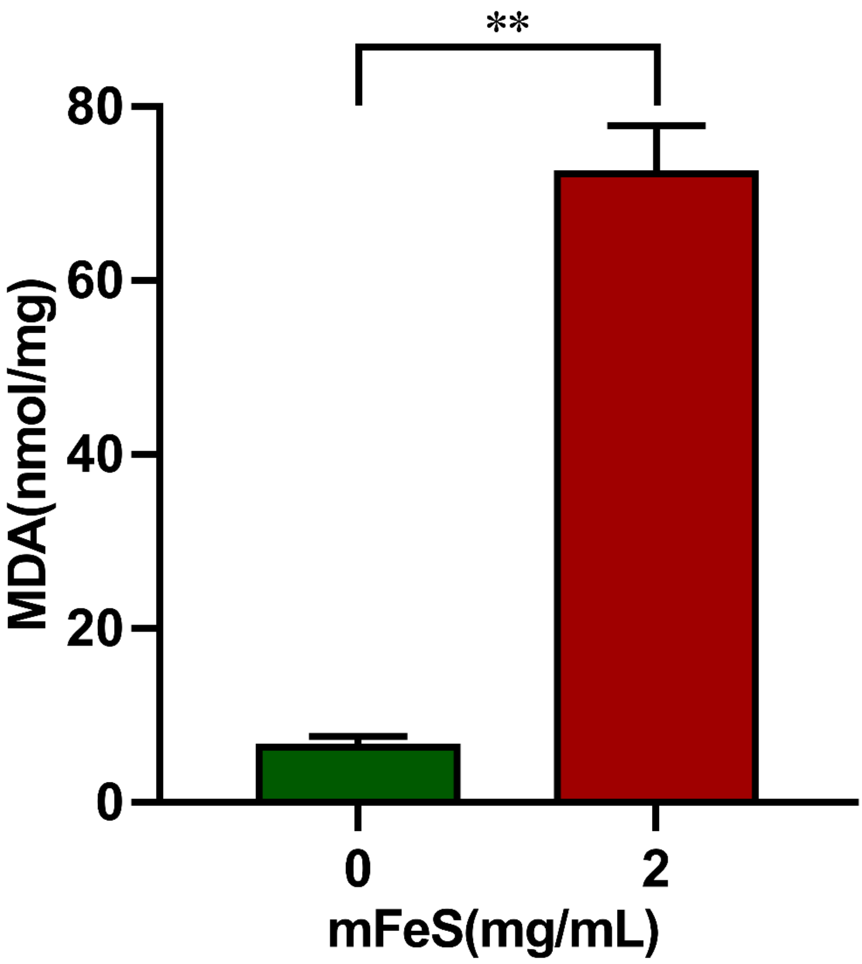

Figure S11 The level of lipid peroxidation of mFeS in liposome. Liposome (4 mg/mL) was treated by mFeS (2 mg/mL) for 2 h. The level of lipid peroxidation (MDA detection) was detected by commercial MDA detection kit according to the manufacturer's instructions. Means ± SD from one of three independent experiments is presented. Student's *t* test is employed to compare the results between different groups. ** $p< 0.01$.

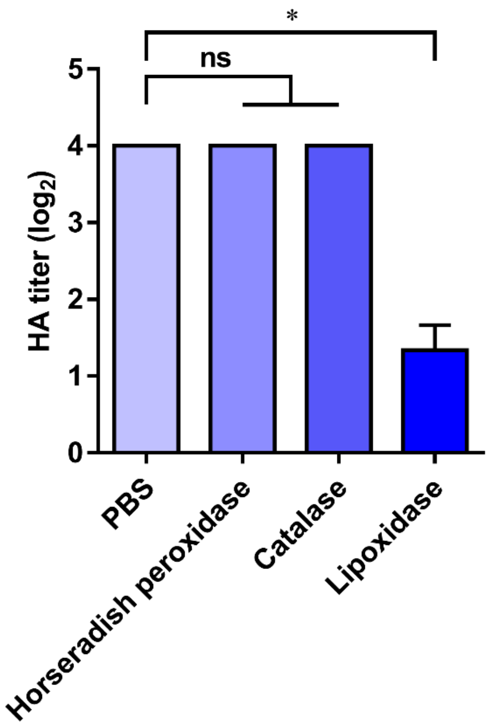

Figure S12 Antiviral effect of lipoxidase, horseradish peroxidase, or catalase on influenza virus. H1N1 virus was treated with 4 mg/mL horseradish peroxidase, catalase, or lipoxidase under 2 h, 37°C, neutral pH. The treated virus was collected to detect viral titers by HA assay. Means ± SD from one of three independent experiments is presented. One-way ANOVA analysis of variance is employed. * $p < 0.05$. *ns,* no significant.

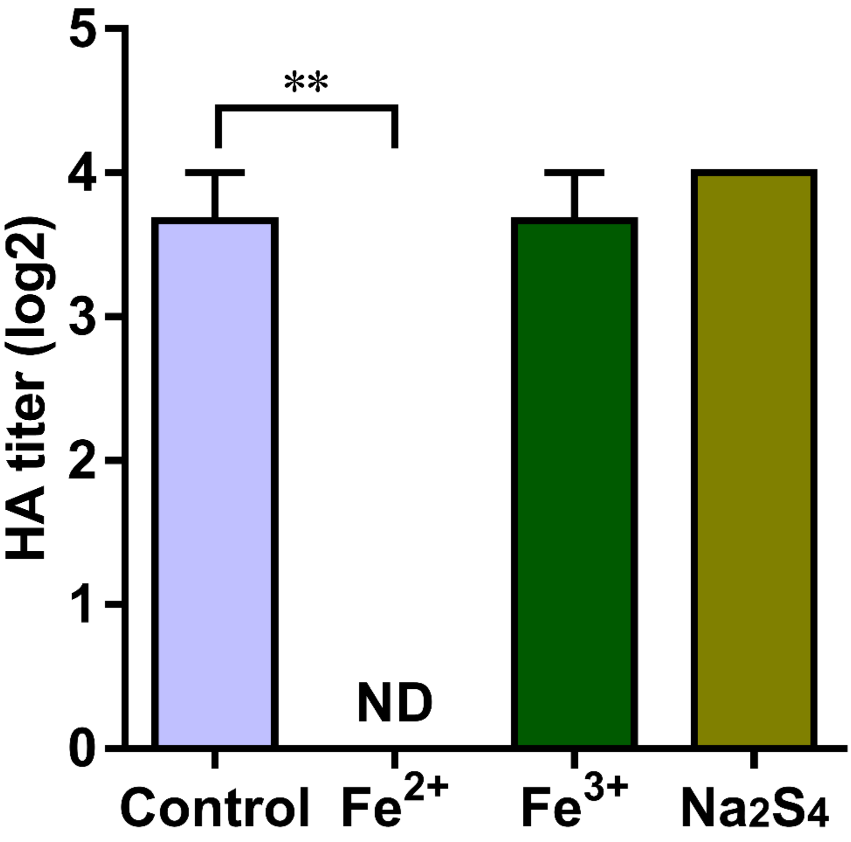

Figure S13 Antiviral activity of main element against influenza virus. H1N1 virus was treated with 200 μM Fe^{2+}, 200 μM Fe^{3+} or 200 μM Na_2S_4 for 2 h. Viral titers were detected by HA assay. Means ± SD from one of three independent experiments is presented. One-way ANOVA analysis of variance is employed. ** $p< 0.01$. *ND*, no detection.

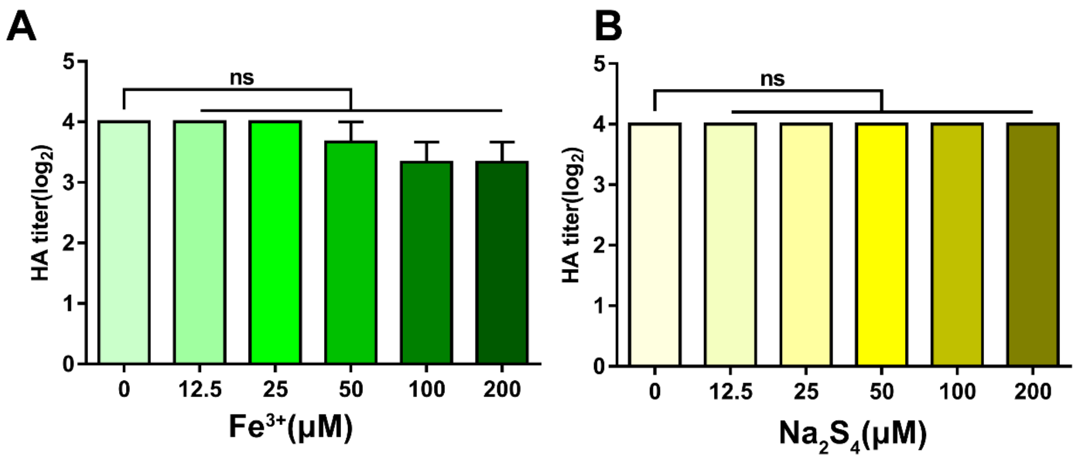

Figure S14 Antiviral activity of variable concentrations of Fe^{3+} or Na_2S_4 against influenza virus. H1N1 virus was mixed with variable concentrations of Fe^{3+} (A) or Na_2S_4 (B) for 2 h. Viral titers were detected by HA assay. Means ± SD from one of three independent experiments is presented. One-way ANOVA analysis of variance is employed. *ns,* no significant.

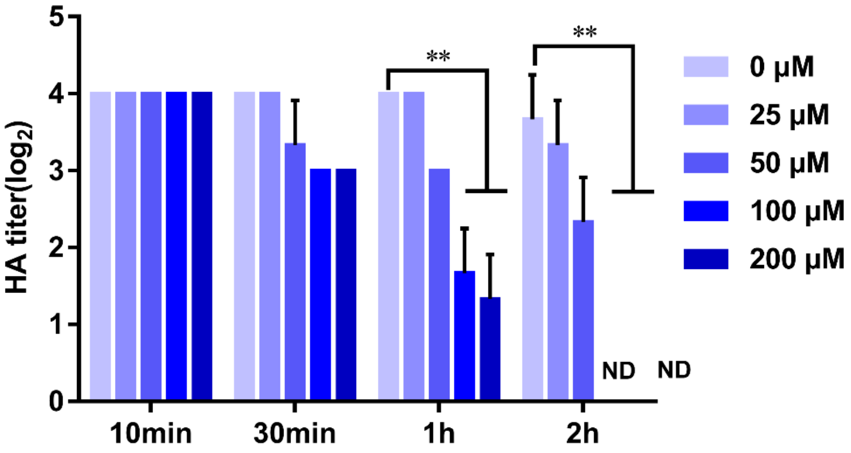

Figure S15 Antiviral activity of variable concentrations of Fe^{2+} against influenza virus. H1N1 virus was mixed with variable concentrations of Fe^{2+} for different time. Viral titers were detected by HA assay. Means ± SD from one of three independent experiments is presented. Two-way ANOVA analysis of variance is employed. ** $p < 0.01$. *ND*, no detection.

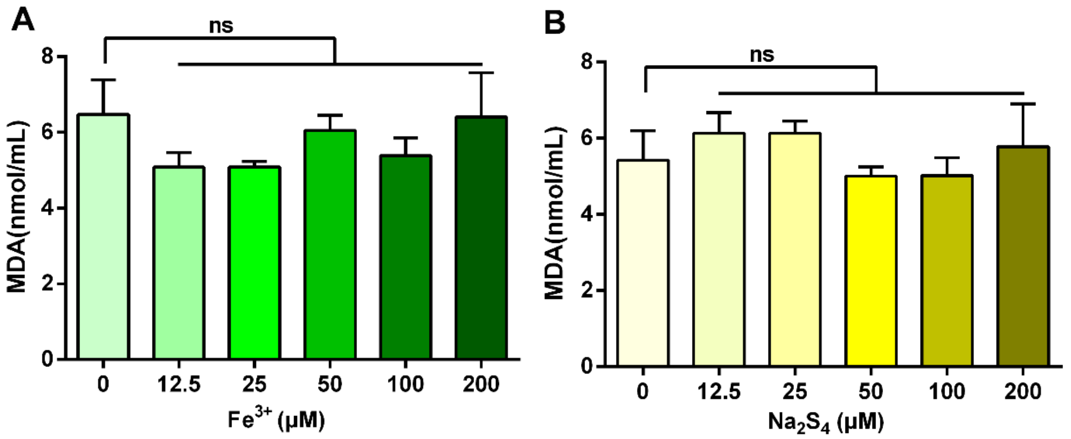

Figure S16 The MDA level of H1N1 virus after treatment with Fe^{3+} and Na_2S_4. H1N1 virus was mixed with variable concentrations of Fe^{3+} (A) or Na_2S_4 (B) for 2 h. The level of viral lipid peroxidation (MDA detection) was detected by commercial MDA detection kit according to the manufacturer's instructions. Means ± SD from one of three independent experiments is presented. One-way ANOVA analysis of variance is employed. *ns,* no significant.

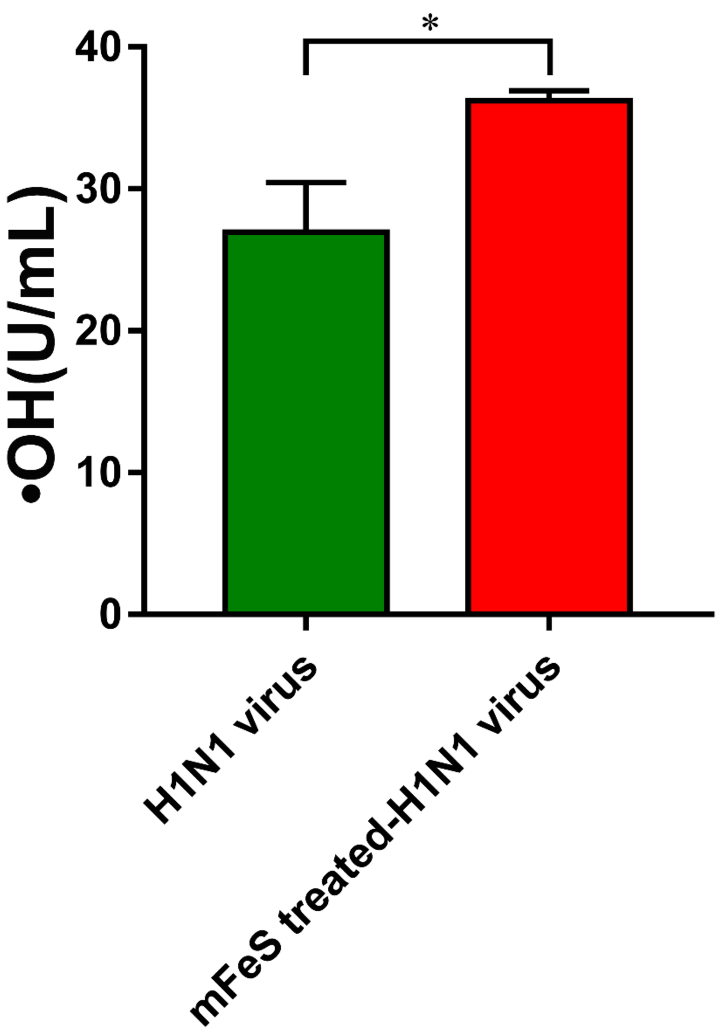

Figure S17 Detection of hydroxyl radicals (•OH). H1N1 virus was treated by mFeS (4 mg/mL) for 2 h. Hydroxyl free radical assay kit (Jiancheng Bioengineering Institute, Nanjing, China) was used to detect the production of hydroxyl radicals (•OH) according to the manufacturer's instruction. Means ± SD from one of three independent experiments is presented. Student's *t* test is employed to compare results between different groups. * $p < 0.05$.

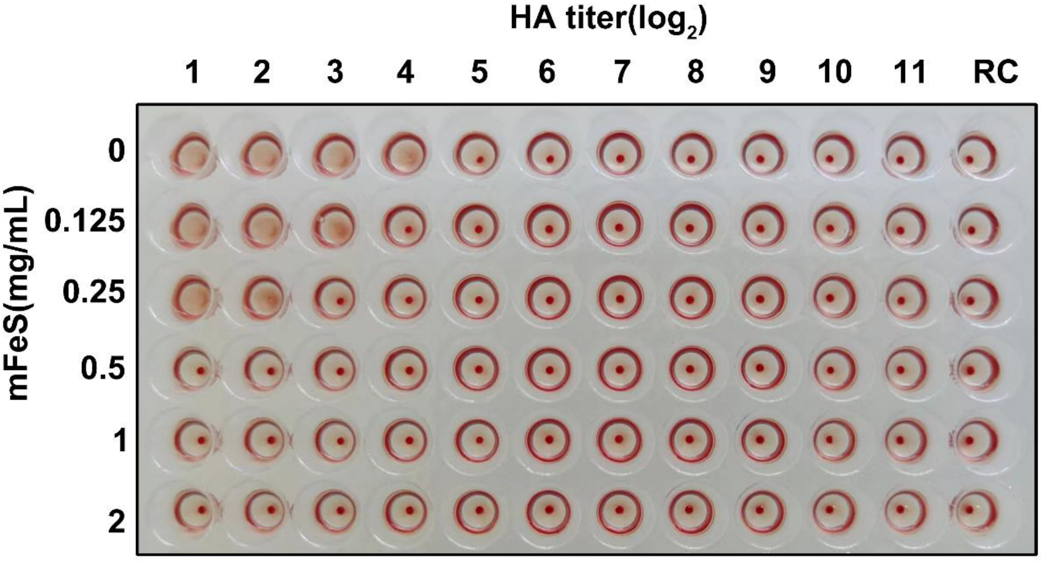

Figure S18 Evaluation of hemagglutination of influenza virus. Different concentrations of mFeS were mixed with H1N1 virus for 2 h. mFeS was pulled down and then supernatant was collected to detect hemagglutination (HA) titers. RC represented the control of red cells. All experiments were repeated in triplicate with a representative image shown.

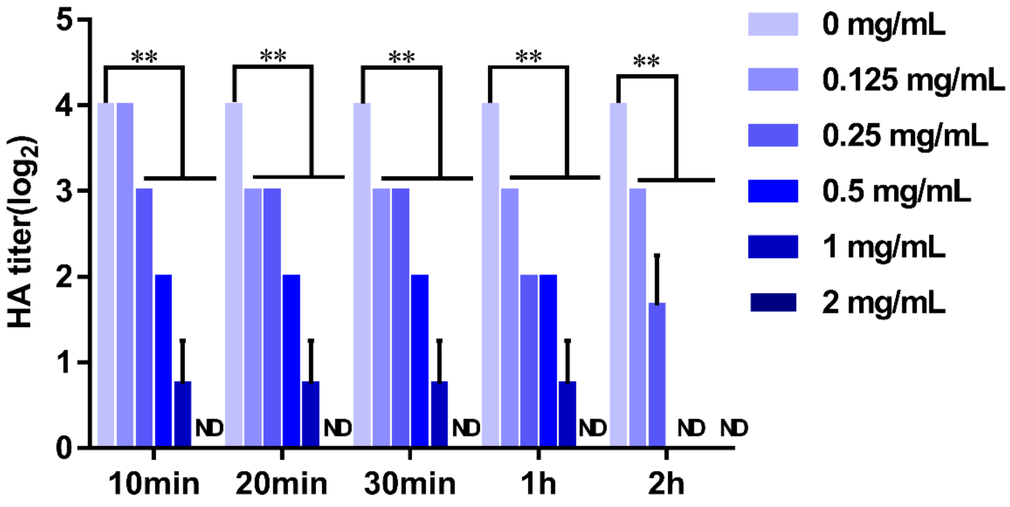

Figure S19 Antiviral activity of variable concentrations of mFeS against influenza virus. H1N1 virus was mixed with variable concentrations of mFeS for different time. mFeS was pulled down by centrifugation and then supernatant was collected to detect viral titers by HA assay. Means ± SD from one of three independent experiments is presented. Two-way ANOVA analysis of variance is employed. ** *p< 0.01*. *ND*, no detection.

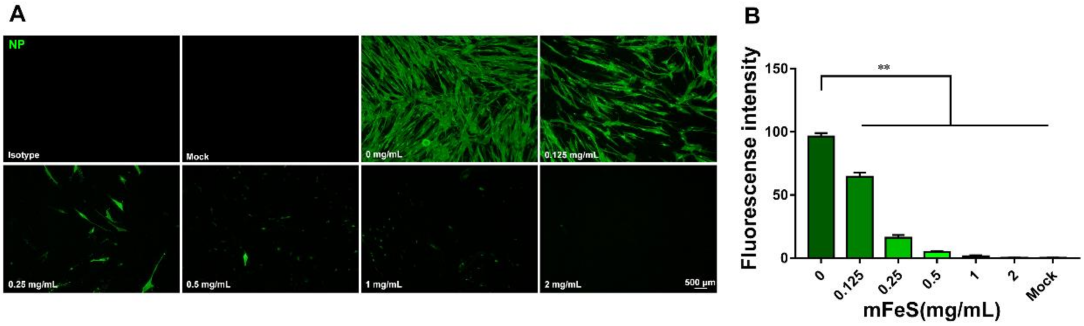

Figure S20 Viral intracellular replication of mFeS-treated H1N1 virus in the CEF cells. CEF cells were infected with mFeS-treated H1N1 virus with a MOI of 1 for 24 h. (A) Cells were fixed to detect viral intracellular replication by immunofluorescence stain of NP protein (green). Scale bar: 500 μm. (B) Fluorescence intensity (green) was measured by imageJ software v1.8. All experiments were repeated in triplicate with a representative image shown. One-way ANOVA analysis of variance is employed. ** $p< 0.01$.

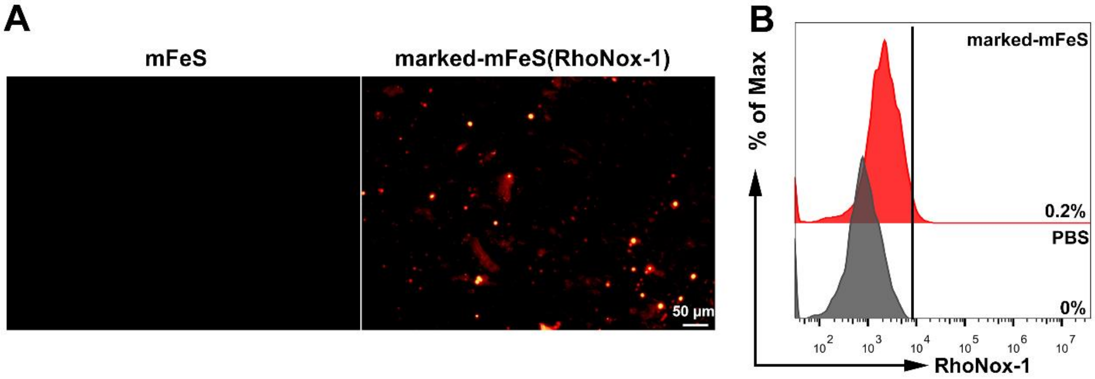

Figure S21 Efficiency of fluorescent probe-marked mFeS entering cells. (A) mFeS was successfully marked by an activatable fluorescent probe (RhoNox-1) which combined with Fe^{2+} irons specially. Scale bar: 50 μm. (B) Cells were collected to detect signals by flow cytometry after incubating with RhoNox-1-marked mFeS for 12 h. All experiments were repeated in triplicate with a representative image shown. Quantification of the flow cytometry resulted as shown in panel.

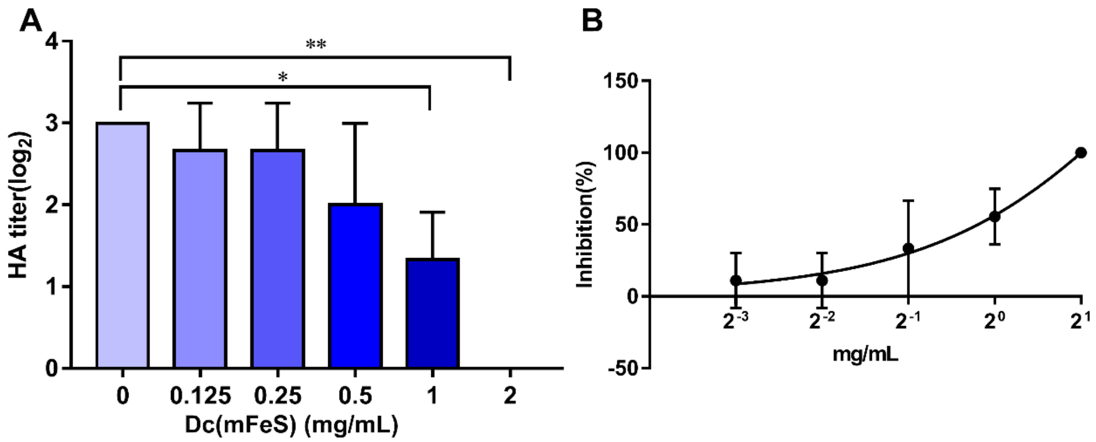

Figure S22 The antiviral therapy of Dc(mFeS) ***in vitro*****.** Variable concentrations of Dc(mFeS) were added to the MDCK cells culture at 12 h.p.i.. (A) HA titer of supernatant was detected after 12 h. (B) The function curve of half maximal effective concentration (EC_{50}) with Dc(mFeS). Means ± SD from one of three independent experiments is presented. One-way ANOVA analysis of variance is employed. * $p< 0.05$, ** $p< 0.01$. *ND*, no detection.

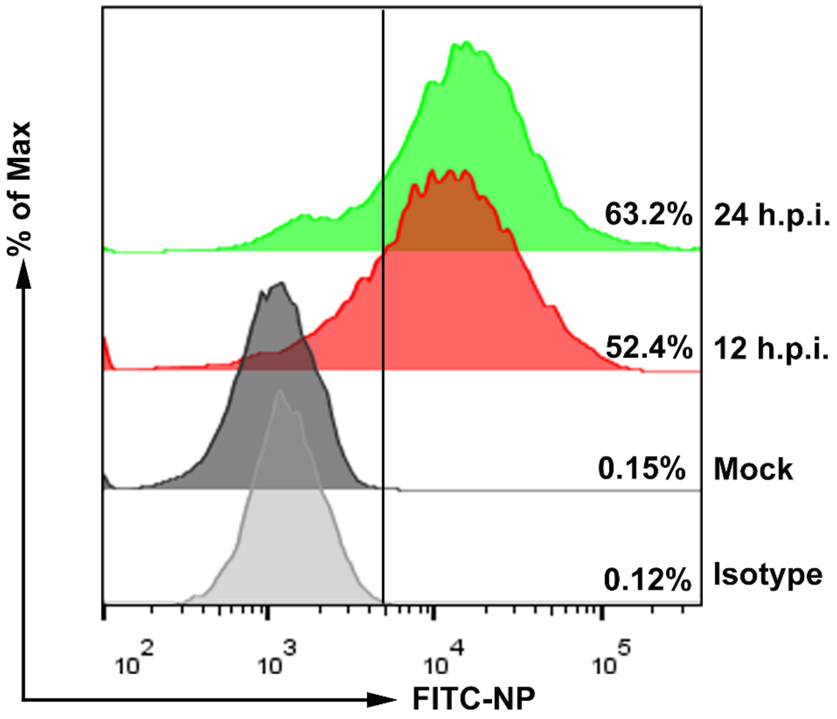

Figure S23 The level of NP protein in H1N1 virus infected-MDCK cells. MDCK cells were infected H1N1 virus at a MOI of 1 for 12 and 24 h. The level of NP protein was detected by flow cytometry. Quantification of the flow cytometry results as shown in panel.

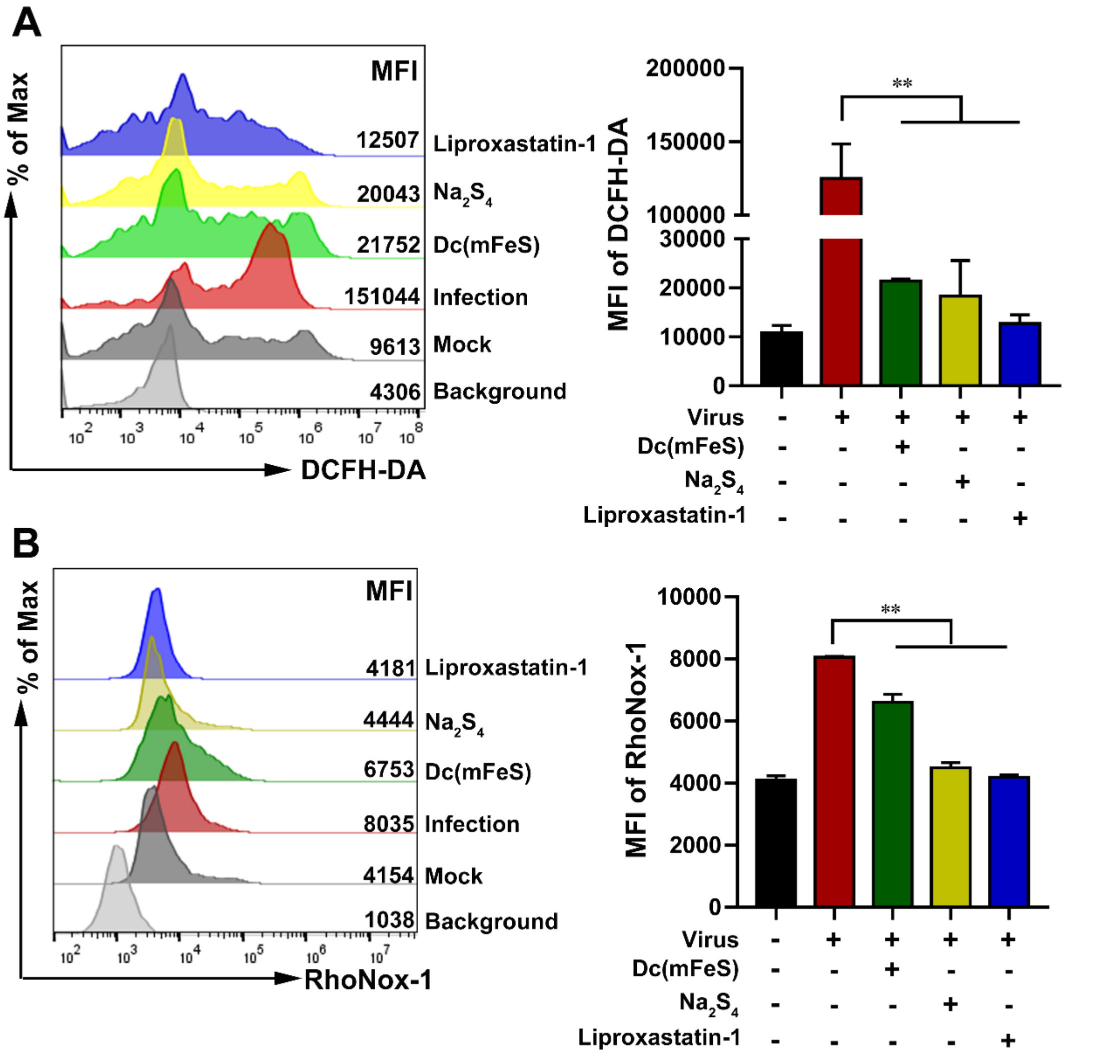

Figure S24 The efficiency of Dc(mFeS) and Na_2S_4 suppressing the intracellular ROS and Fe^{2+} level after H1N1 virus infection. (A-B) MDCK cells were infected with H1N1 virus at a MOI of 1. Dc(mFeS) (4 mg/mL, 500 μL), ferroptosis inhibitor (Liproxastatin-1, 2 μM) or Na_2S_4 (500 μM) were added to the infected cells at 12 h.p.i., respectively. (A) Cells were collected after 12 h to detect the intracellular ROS level by using a 2',7'-dichlorofluorescin diacetate (DCFH-DA) fluorescent probe. (B) Cells were collected after 12 h to detect the intracellular Fe^{2+} level by using fluorescent probe (RhoNox-1). Quantification of the flow cytometry resulted as shown in panel. Means ± SD from one of three independent experiments is presented. One-way ANOVA analysis of variance is employed. * *p< 0.05*, ** *p< 0.01*.

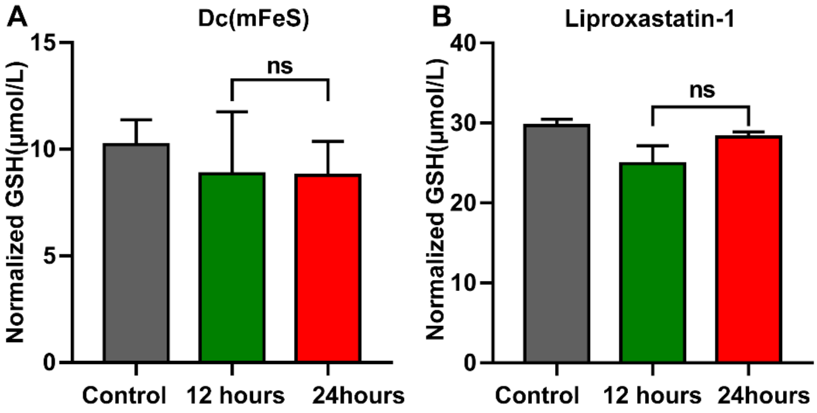

Figure S25 The influence of Dc(mFeS) and Liproxastatin-1 on normalized GSH level. Dc(mFeS) or ferroptosis inhibitor (Liproxastatin-1) was added to the normally cultured MDCK cells. (A) The mFeS-treated cells were collected to detect normalized GSH after12 h and 24 h. (B) The Liproxastatin-1-treated cells were collected to detect normalized GSH after 12 h and 24 h. Means ± SD from one of three independent experiments is presented. One-way ANOVA analysis of variance is employed. *ns,* no significant.

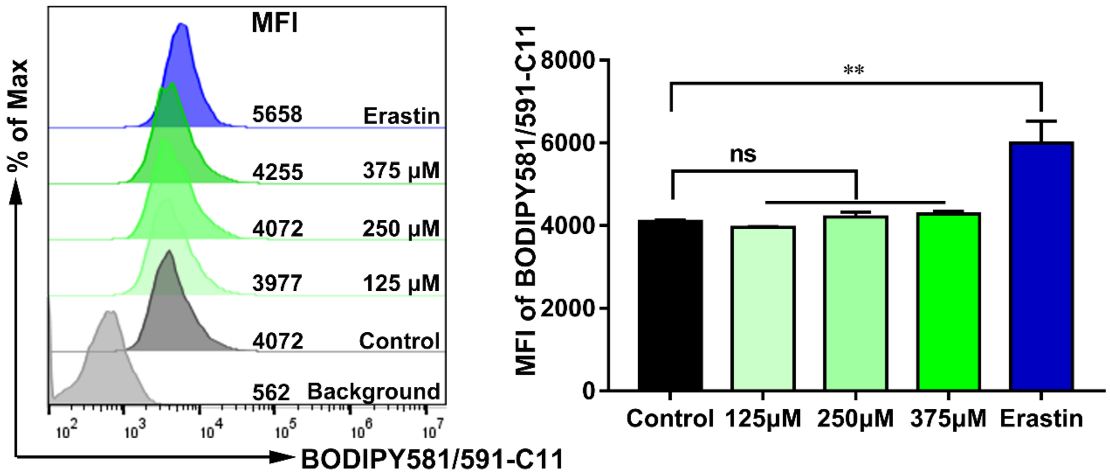

Figure S26 The level of lipid peroxidation in MDCK cells treated by Fe^{2+}. MDCK cells were treated with different concentrations of Fe^{2+} and Erastin (5 μM). Cells were collected after 12 h to detect the lipid peroxidation level using BODIPY581/591-C11 probe. Quantification of the flow cytometry resulted as shown in panel. Means ± SD from one of three independent experiments is presented. One-way ANOVA analysis of variance is employed. *ns,* no significant. ** $p< 0.01$.

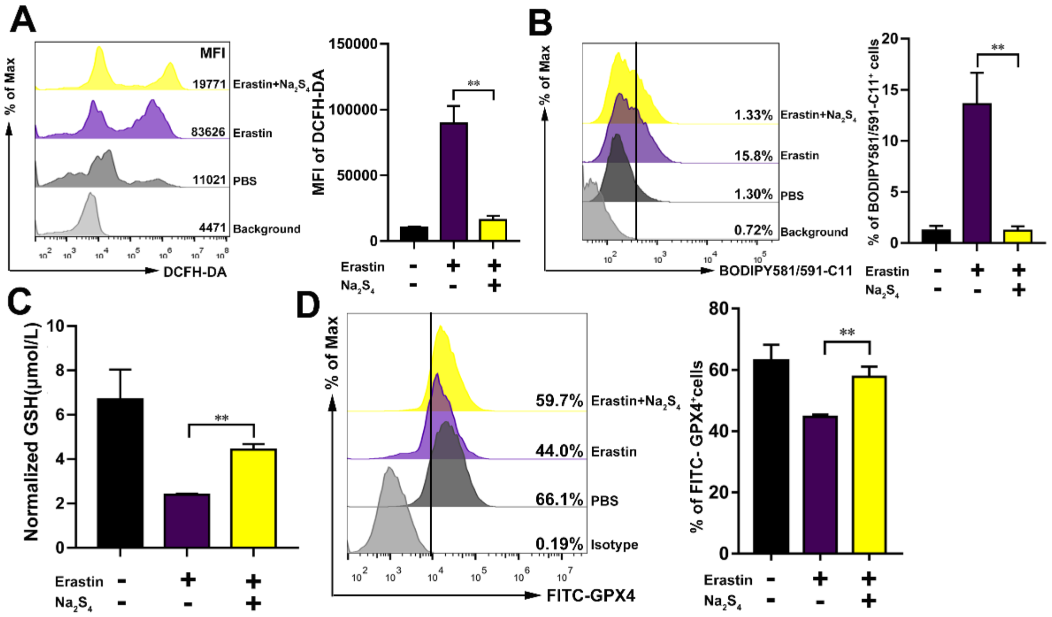

Figure S27 The efficiency of Na2S4 suppressing cellular ferroptosis. MDCK cells were added 5 μM Erastin to induce ferroptosis, and then Na_2S_4 (500 μM) was added after 12 h. (A) The intracellular ROS level was detected by using a 2',7'-dichlorofluorescin diacetate (DCFH-DA) fluorescent probe after 24 h. (B) BODIPY581/591-C11 was used as the probe to detect lipid peroxide after 24 h by flow cytometry. (C) Normalized GSH was detected by GSH detection kit after 24 h. (D) Cells were collected to detect the expression of GPX4 by flow cytometry after 24 h. Quantification of the flow cytometry resulted as shown in panel. Means ± SD from one of three independent experiments is presented. One-way ANOVA analysis of variance is employed. * $p < 0.05$, ** $p < 0.01$.

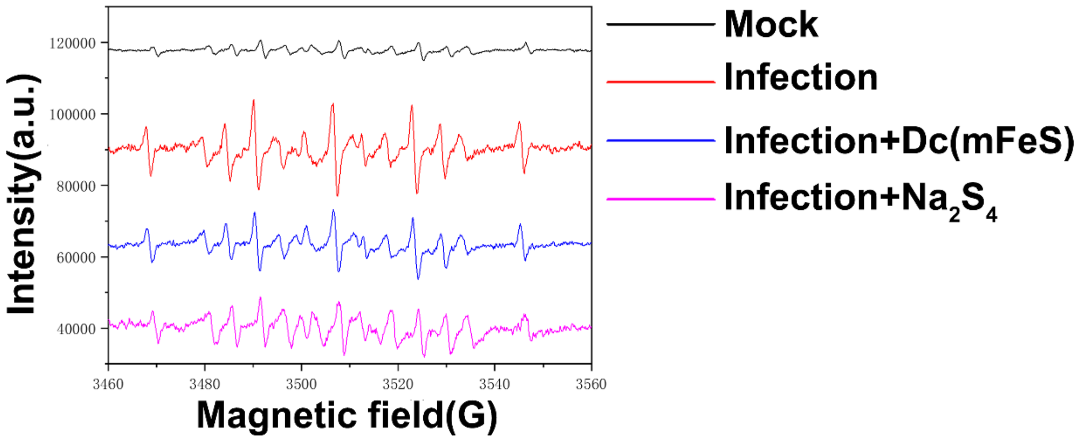

Figure S28 EPR analysis of MDCK cells treated with Dc(mFeS) and Na_2S_4 during the viral infection. Dc(mFeS) (4 mg/mL, 500 μL) or Na_2S_4 (500 μM) was added to the H1N1 virus-infected MDCK cells at 12 h.p.i. Cells were collected to detect the free radical levels after 12 h using EPR.

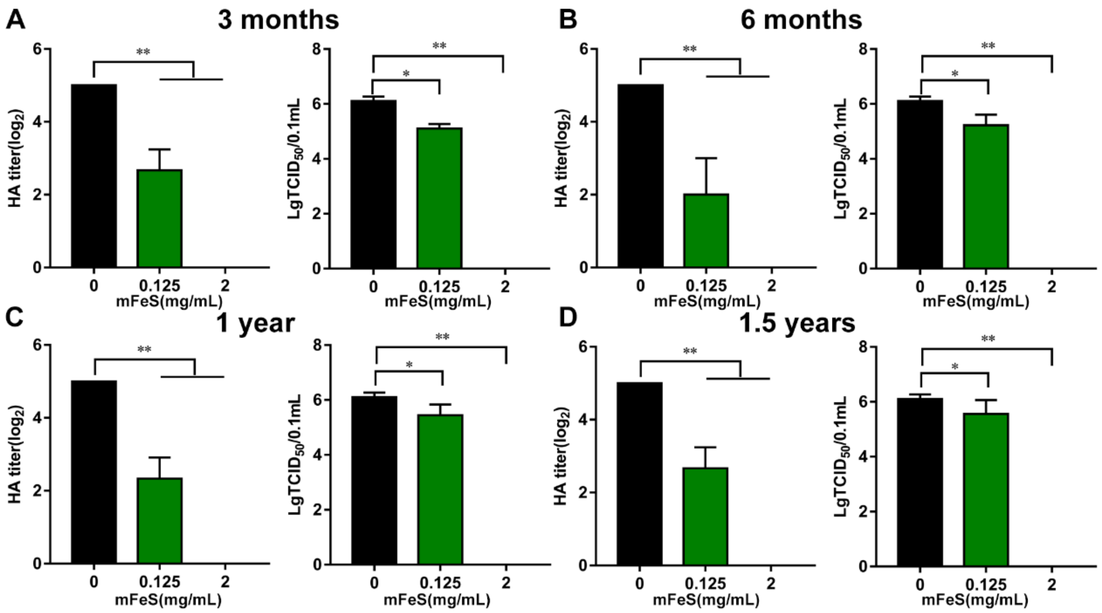

Figure S29 Antiviral ability of mFeS with a long-term storage. mFeS was stored for 3 months (A), 6 months (B), 1 year (C) and 1.5 years (D) at room temperature. H1N1 virus was treated with stored mFeS for 2 h. mFeS was pulled down by centrifugation and then supernatant was collected to detect viral titers by HA assay and $TCID_{50}$. Means ± SD from one of three independent experiments is presented. One-way ANOVA analysis of variance is employed. * $p< 0.05$, ** $p< 0.01$. *ND*, no detection.

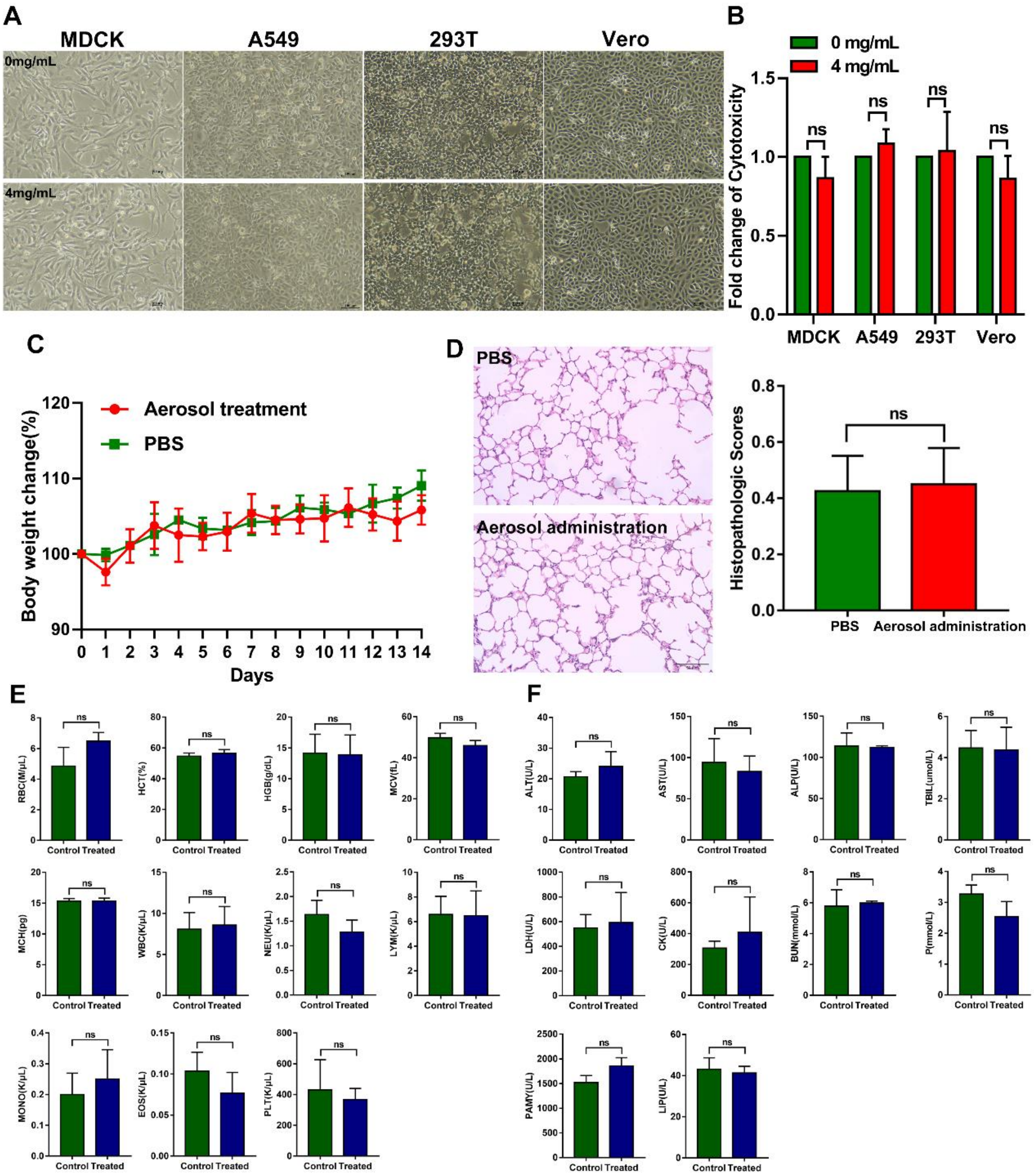

Figure S30 Biosafety evaluation of mFeS&Dc *in vitro* and *in vivo*. Variable concentrations of mFeS&Dc were added during the MDCK, A549, 293T and Vero cells. (A) Cellular morphology was observed by electronic microscope after treatment. Scale bar: 100 μm. (B) Cell counting kit-8 was used to detect cytotoxicity of mFeS&Dc on MDCK, A549, 293T and Vero cells. (C-D) BALB/c mice were treated with 2 mL mFeS&Dc (50 μg/mL) through aerosol administration. Atomization treatment was performed every 6 h for 7 days. (C) Morbidity was evaluated by monitoring weight changes over a 14-day period and was plotted as a percentage of the weights on the day of inoculation (day 0) (n = 5). (D) Lung tissues of the treated mice (n = 3) were collected

after 14 days. Representative histopathological changes and histopathologic scores in H&E (hematoxylin and eosin)-stained lung tissues. Scale bar: 50 μm. The blood routine (E) and blood biochemical (F) indicators of mice which was treated with mFeS&Dc (n = 3). RBC, Red blood cells; HCT, Red blood cell specific volume; HGB, Hemoglobin; MCV, Mean corpuscular volume; MCH, Mean corpuscular hemoglobin; WBC, White blood cell; NEU, Neutrophil; LYM, Lymphocyte; MONO, Monocyte; EOS, Eosinophils; PLT, Platelet; ALT, Alanine transaminase; AST, Aspartate transaminase; ALP, Alkaline phosphatase; TBIL, Total bilirubin; LDH, Lactic de-Hydrogenase; CK, Creatine kinase; BUN, Blood urea nitrogen; P, Phosphorus; PAMY, Pancreatic amylase; LIP, Lipase. Means ± SD from one of three independent experiments is presented. Student's *t* test is employed to compare results between different groups. *ns,* no significant.

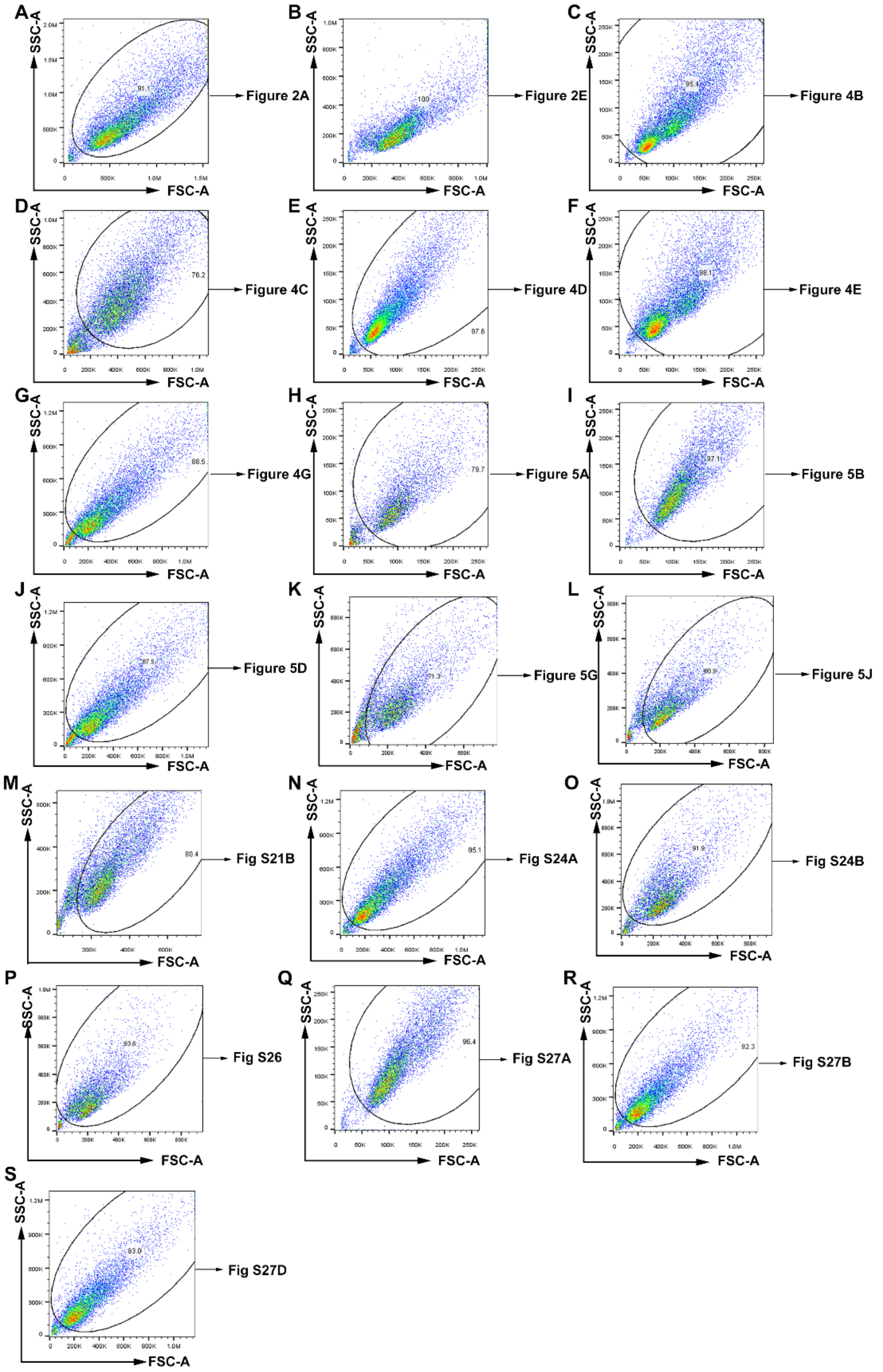

Figure S31 Schematic gating strategy of flow cytometry. A is related to Figure 2A. B is related to Figure 2E. C is related to Figure 4B. D is related to Figure 4C. E is related to Figure 4D. F is related to Figure 4E. G is related to Figure 4G. H is related to Figure 5A. I is related to Figure 5B. J is related to Figure 5D. K is related to Figure 5G. L is related to Figure 5J. M is related to Fig S21B. N is related to Fig S24B. O is related to

Fig S24B. P is related toFig S26. Q is related to Fig S27A. R is related to Fig S27B. S is related to Fig S27D.

Table S1 Kinetic parameters of mFeS.

Substrate	K_M (mM)	V_{max} (nM/s)
H_2O_2 + TMB	373.1	538.1
TMB	616.5	949.5

Table S2 The strains of the influenza *A/B* virus used in this study.

Viruses	Subtype/Clade
A/PR/8/34 H1N1	H1N1
A/Swine/Jiangsu/48/2010	H1N1
A/duck/Wuxi/2/2013	H2N2
A/Duck/Eastern China/866/2003	H3N2
A/Duck/Eastern China/160/2002	H4N6
A/Chicken/Jiangsu/XZXY/2013	H5N2
A/Duck/Eastern China/164/2002	H6N2
A/Chicken/Eastern China/JD/2017	H7N9
A/Duck/Eastern China/01/2005	H8N4
A/Duck/Eastern China/01/2000	H9N2
A/Duck/Eastern China/488/2003	H10N3
A/Duck/Eastern China/05/2005	H11N2
B/Human/Jiangsu/10/2022	Victoria
